# Low‐Dose H_2_O_2_ Priming Improves Performance Under Simulated Marine Heatwave Conditions in a Coastal Bivalve

**DOI:** 10.1002/gch2.70123

**Published:** 2026-06-14

**Authors:** Chiara Mascitelli, Giovanna Monticelli, Alessandro Nardi, Giulia Dalla Rovere, Graziano Rilievo, Ilaria Bernardini, Nicole Macrì, Deborah Cesaroni, Valentina Tavolazzi, Gabriele Andreatta, Tomaso Patarnello, Luca Bargelloni, Luca Peruzza, Maura Benedetti, Massimo Milan

**Affiliations:** ^1^ Department of Comparative Biomedicine and Food Science University of Padova Legnaro Padova Italy; ^2^ Galilean School of Higher Education University of Padova Padova Italy; ^3^ Department of Life and Environmental Sciences Marche Polytechnic University Ancona Italy; ^4^ National Biodiversity Future Center Palermo Italy

**Keywords:** chemical priming, climate change adaptation, environmental stress, hydrogen peroxide, marine heatwaves, stress memory

## Abstract

Marine heatwaves are intensifying under climate change, imposing thermal extremes on marine ecosystems. Priming, a process in which prior sublethal exposure shapes responses, is increasingly recognized as a key mechanism underlying tolerance to environmental variability. While widely studied in plants, chemical priming remains unexplored in animals. Here, we tested whether hydrogen peroxide (H_2_O_2_), acting as a redox signal, can induce a primed physiological state modulating response to simulated marine heatwaves in the Manila clam (*Ruditapes philippinarum*), an ecologically and economically important bivalve species. Clams were exposed to chemical, thermal, or combined priming treatments and subsequently challenged with a controlled heatwave. H_2_O_2_ priming emerged as an effective and well‐tolerated treatment, with primed clams exhibiting faster burrowing during heatwave exposure and minimal transcriptomic perturbation. Microbiota analyses revealed transient shifts and reduced relative abundance of opportunistic taxa (including *Vibrio* and *Tenacibaculum*) during and after heatwave exposure. A long‐term field trial under natural summer conditions did not reveal detectable adverse effects on the measured traits over several months following priming. Overall, these findings identify low‐dose H_2_O_2_ priming as a potential mechanism shaping stress responses in marine bivalves under simulated heatwave conditions.

## Graphical abstract

Low‐dose hydrogen peroxide priming shapes Manila clam responses to simulated marine heatwaves, which showed faster burrowing behavior, limited transcriptional disruption, and reduced opportunistic microbiota. A long‐term field trial confirms no detectable adverse effects, supporting redox‐based chemical priming as a feasible resilience strategy in bivalve aquaculture.

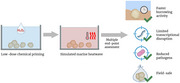

## 1 Introduction

Climate change is reshaping marine environments by increasing both mean temperatures and the frequency of extreme thermal events. Among these, marine heatwaves (MHWs), defined as prolonged periods of anomalously high seawater temperature, have intensified markedly over the past century and are emerging as recurrent features of coastal ecosystems [1]. These extreme thermal events profoundly impact marine ecosystems, triggering biological responses from individuals to entire communities [2], imposing strong selective pressures on marine ectotherms, particularly sessile invertebrates [3]. Recurrent thermal extremes impair organismal performance across multiple levels of biological organization, from metabolism and behavior to cellular homeostasis, and are frequently associated with mass mortality events in coastal systems [4, 5]. Importantly, thermal stress also alters host‐associated microbial communities, highlighting that responses to marine heatwaves involve the holobiont rather than the host alone [6, 7]. As a consequence, tolerance to marine heatwaves depends on the ability of organisms to express adaptive plasticity and retain stress memory, allowing them to modulate physiological and ecological responses to repeated thermal challenges [8, 9].

A species that is particularly sensitive to such changes is the Manila clam (*Ruditapes philippinarum*), a bivalve mollusk of great economic and ecological importance. Globally, more than 4,000 million tons of Manila clams are harvested each year in coastal lagoons and deltas, sustaining a major aquaculture sector [10]. This species is native to the Indo‐Pacific region, but it has been introduced worldwide for aquaculture purposes. It is now one of the most farmed shellfish species, playing a key role in the economic income of coastal communities. Beyond its economic value, the Manila clam contributes to ecosystem functioning through bioturbation, water filtration, and carbon sequestration, thereby influencing nutrient cycling and sediment structure [11, 12]. In terms of carbon emissions, bivalves like Manila clam are among the most sustainable food sources, with extremely low greenhouse gas footprints compared to terrestrial protein sources [13, 14]. However, this benthic species is severely threatened by climate change‐related extreme events, in particular MHWs, which tend to be particularly intense in shallow coastal habitats where most clam farming occurs. Notably, even when not directly lethal, MHWs can compromise animal physiology and stress tolerance [15]. Therefore, it is paramount to develop strategies to mitigate the effects of climate change.

In crops, a powerful approach to enhance stress resilience is priming, which involves controlled exposure to sublethal stress that “prepares” an organism to mount a more robust defense against subsequent lethal challenges, through a process of stress memory [16]. More broadly, priming can be viewed as a general biological mechanism of adaptive plasticity, whereby prior exposure to a sublethal stimulus alters organismal physiological states and modulates responses to recurrent environmental extremes. This process is well established in plants and encompasses multiple modalities, including thermal priming, which enhances thermotolerance and fortifies antioxidant defenses, and chemical priming, in which defined agents pre‐activate protective signaling pathways involved in reactive oxygen species (ROS) detoxification, osmoprotection, and protein homeostasis [17, 18, 19].

For sessile animals like bivalves, which can only to a limited extent rely on avoidance behavior to survive in fluctuating environments, priming is a promising and increasingly investigated avenue. Encouraging results have recently been reported for thermal priming in molluscs [20, 21, 22, 23], demonstrating its capacity to modulate physiological performance and stress responses under heat exposure, although the literature on this topic remains relatively limited. In contrast, despite widespread use in plants to enhance resistance to different abiotic stresses [19, 24], chemical priming has not yet been experimentally tested in animal systems, to the best of our knowledge. Among the molecular processes underlying priming responses, redox signaling plays a central role in mediating stress perception and stress memory across taxa [25, 26]. Reactive oxygen species (ROS), once considered merely harmful by‐products of metabolism, are now recognized as key signaling molecules involved in the regulation of cellular homeostasis, metabolic reprogramming, and stress‐responsive pathways [27, 28]. Transient and tightly controlled changes in cellular redox state can therefore act as informational cues, enabling organisms to adjust physiological responses to environmental stressors. Within this framework, hydrogen peroxide (H_2_O_2_) represents a particularly relevant redox signal due to its relative stability and conserved role in stress‐related signaling pathways. Reactive oxygen species, including H_2_O_2_, are naturally present in marine environments and are generated through both metabolic and environmental processes. In this context, H_2_O_2_ can be used as a controlled experimental stimulus to induce redox‐mediated priming, rather than to replicate direct environmental exposure. At low concentrations, H_2_O_2_ functions as a signaling molecule capable of modulating transcriptional, metabolic, and antioxidant responses, and has been widely employed to experimentally induce primed physiological states in plants exposed to abiotic stress [24, 29, 30, 31, 32]. However, whether redox‐active molecules such as H_2_O_2_ can induce a primed physiological state in animal species remains unknown.

Here, we hypothesized that redox‐mediated priming could represent a physiological mechanism through which marine bivalves modulate responses to thermal extremes. Specifically, we exposed juvenile Manila clams to chemical, thermal, or combined priming treatments and subsequently challenged them with a simulated marine heatwave to test whether low‐dose H_2_O_2_ priming improves responses under thermal stress conditions. Responses were evaluated across complementary levels of biological organization, including biometric traits, behavior, transcriptomic profiles, and host‐associated microbiota dynamics. In parallel, a field experiment under natural summer conditions was conducted to assess the persistence and ecological relevance of priming effects. Overall, this study provides the first experimental evidence that chemical priming can modulate organismal and holobiont responses to marine heatwaves, offering mechanistic insight into how marine ectotherms may cope with increasingly frequent thermal extremes under climate change.

## 2 Methods

### 2.1 Animals and Experimental Procedures

**FIGURE 1:**
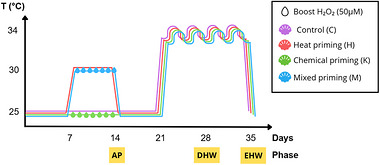
Diagram showing the experimental design depicting the various priming treatments, in terms of duration and water temperature, and the subsequent simulated heatwave. (AP: after priming; DHW: during heat wave; EHW: end heat wave).

Juvenile Manila clams (*Ruditapes philippinarum*; mean shell length 20.13 ± 1.48 mm) were obtained from a commercial hatchery (SATMAR, France). Full tank configuration and husbandry details are provided in Section S1. Briefly, after a short depuration period, clams were maintained in 60‐L bi‐chamber aerated tanks under controlled salinity (28.5–30.5 PSU) and with recirculating artificial seawater. Animals were fed daily with a commercial microalgal mixture, which, while necessary to maintain physiological condition, may represent a potential source of variability due to indirect effects on feeding dynamics. Temperature was monitored continuously and regulated using programmable thermoregulators for thermal priming and to generate heatwave profiles as described in ref. [22]. Figure 1 illustrates the experimental setup. Four experimental conditions were set up in triplicate tanks (12 tanks in total): heat‐primed (H), chemical‐primed (K), mixed‐primed (M), and control (C). After seven days of acclimation at 25°C, clams underwent a 7‐day priming phase: H, constant 30°C; K, 50 µm H_2_O_2_ (daily replenished); M, constant 30°C + 50 µm H_2_O_2_; C, constant 25°C.

H_2_O_2_ concentration was chosen based on the results of a pilot test (data not shown) comparing 10 and 50 µm, with the latter showing a stronger effect in inducing a physiological response while remaining non‐lethal. The selected concentration is intended to act as a controlled redox stimulus to induce priming, rather than to reproduce environmentally occurring H_2_O_2_ levels. H_2_O_2_ concentrations in tank water were monitored using a spectrophotometric assay to assess its persistence under experimental conditions (see details in Appendix S1).

Following priming and a seven‐day resting period under control conditions, the four treatment groups were exposed to a 14‐day challenge, consisting of a simulated MHW orchestrated by thermoregulators. The MHW water temperature followed a 24‐h stepped profile with linear ramps between plateaus: 0–10 h: 31°C; 11–13 h: intermediate step at 32°C; 13–18 h: ramp up toward 34°C; 18–21 h: ramp down toward 32°C; 21–22 h: intermediate step at 32°C; 22–24 h: 31°C. This temperature profile, mirroring the intensity and duration of recent natural heatwaves in the Venice Lagoon [15], was designed to impose a strong but overall non‐lethal thermal challenge, ensuring full animal availability for all downstream analyses.

To assess their safety under natural conditions, priming treatments were also applied to a second batch of clams, which was seeded immediately after priming in the Venice Lagoon within a farming site that experienced serious HW‐related mortality events in the past [33]. Clams were individually tagged, placed in underwater cages, and monitored over the summer for temperature exposure, survival, and condition index.

### 2.2 Condition Index

Condition indices (CI) were calculated as the ratio of dry tissue mass to dry shell mass (CI = dry tissue weight/dry shell weight) in agreement with [34]. For each replicate tank, three clams were randomly sampled (9 clams per condition per time point). Sampling occurred at two stages of the experiment: after seven days (DHW) and at the end of the heatwave (EHW). Outcomes were analyzed using a two‐way ANOVA to evaluate the effects of Group, Stage, and their interaction (Group x Stage), after checking for normal distribution and heteroscedasticity.

CIs were also calculated for the second batch of clams after recovering them from the field. A total of 15 clams per group (five from each of the three cages) were randomly sampled per group. Differences in CI among groups were tested using a linear mixed‐effects model with Group as a fixed factor and Cage as a random blocking factor, to account for the non‐independence of individuals within the same cage. Model assumptions were checked by inspecting residual plots.

### 2.3 Burrowing Test

Burrowing behavior was assessed at three time points (AP, DHW, EHW) using 15 clams per treatment. Full methodological details are provided in the Supporting Information. Briefly, clams were placed on sediment in a plastic container, and the time to complete burial was recorded over 30 min. Burrowing dynamics were analyzed using Kaplan‐Meier curves and log‐rank tests, complemented by Cox models to compare burial rates among treatments. The proportion of burrowed clams at specific time points was additionally tested using binomial Generalized Linear Models (GLMs).

### 2.4 Transcriptomic Analysis

Digestive glands were sampled at AP, DHW, and EHW and processed for RNA‐seq. Full methodological details and QC metrics are provided in the Supporting Information. Briefly, total RNA was extracted and quality‐checked before library preparation and sequencing (Illumina NovaSeq X, PE150). Reads were processed with the nf‐core/rnaseq pipeline and quantified with RSEM [35]. Gene‐level counts were subjected to low‐expression filtering using the filterByExpr function from edgeR (min. count = 5, min. total. count = 15, min. prop = 0.7), followed by variance‐stabilizing transformation (VST) using DESeq2. Principal component analysis (PCA) and permutation‐based multivariate analysis of variance (PERMANOVA) were performed separately for each time point using the vegan package (adonis function, vegan v2.7‐1) to assess the effect of treatment on overall transcriptomic composition. In addition, to assess the effect of the experimental phases on the baseline transcriptomic profile, PCA and PERMANOVA were performed on the control group, and to identify which time points differed significantly from each other, pairwise PERMANOVA was subsequently performed using the pairwise.adonis2 function. Differential expression analysis was performed in edgeR [36] with RUVSeq normalization, applying FDR ≤ 0.1 and |log_2_FC| ≥ 1.5. All differential expression analyses compared each priming treatment (H, K, M) against the non‐primed control group (C). Functional enrichment and Gene Set Enrichment Analysis (GSEA) were conducted using clusterProfiler [37]. For GSEA, significance was assessed using an FDR cutoff of 0.2, consistent with commonly adopted thresholds for exploratory pathway‐level analyses, where relatively relaxed FDR thresholds (up to ∼0.25) are often considered acceptable in exploratory analyses (Subramanian et al., 2005) [38]. Sequences are available in NCBI (BioProject ID: PRJNA1391265).

### 2.5 Microbiota Analysis

Bacterial communities were profiled by sequencing the V3‐V4 region of the 16S rRNA gene from cDNA generated from the same RNA used for transcriptomics. Briefly, reads were processed with the nf‐core/ampliseq pipeline and analyzed in phyloseq [39] after filtering low‐abundance taxa. In detail, taxa with fewer than 10 total reads or present in fewer than 3 samples were removed to reduce the impact of low‐abundance and potentially spurious taxa, thereby improving the robustness of downstream community analyses. Alpha diversity (Shannon, Observed Richness) was compared among treatments via Kruskal‐Wallis tests, while beta diversity (Bray‐Curtis, weighted UniFrac) was assessed through Principal Coordinates Analysis (PCoA) and Permutational Multivariate Analysis of Variance (PERMANOVA) with dispersion checks. All analyses tested each priming treatment (H, K, M) against the non‐primed control (C). Differentially abundant taxa were identified using DESeq2 [40]. Sequences are available at the NCBI (BioProject ID: PRJNA1391265). Details regarding each methods section, including H_2_O_2_ kinetics, are reported in Appendix S1.

## 3 Results and Discussion

### 3.1 H_2_O_2_ Stability and Decay Dynamics

H_2_O_2_ decay followed first‐order kinetics and was strongly influenced by both the presence of clams and temperature (Appendix S1 and Figures S5–S9). In seawater without clams, the degradation rate constant was k = 0.19 ± 0.03 h^−1^, with H_2_O_2_ becoming undetectable after ∼20 h. In the presence of clams, decay was markedly faster (k = 0.41 ± 0.08 h^−1^), with H_2_O_2_ no longer detectable after 5–6 h. At 30°C, degradation rates further increased (k = 0.80 ± 0.05 h^−1^), approximately doubling compared to room temperature conditions, indicating a strong temperature dependence of H_2_O_2_ stability. Together, these results indicate that H_2_O_2_ persistence is strongly reduced under elevated temperature conditions. In the mixed treatment, this may have limited the contribution of H_2_O_2_ to redox‐mediated priming, potentially resulting in a response more similar to thermal priming alone.

### 3.2 Priming Treatments Do Not Impact on Condition Index and Viability

The condition index (CI) is a widely used physiological biomarker of bivalve health, as it reflects both nutritional status and exposure to physiological stress [41, 42, 43]. As the overarching goal is to develop effective strategies to mitigate the effects of thermal stress in coastal bivalves, we first assessed whether priming impacted physiological condition and survival. In line with our expectations, CI did not differ significantly among treatments at either sampling point (DHW: *p* = 0.478; EHW: *p* = 0.193), indicating that neither thermal, nor chemical, nor combined priming strategies produced detectable positive or negative effects on overall clam condition during the controlled heatwave experiment. Consistently, survival remained high and comparable among groups (*p* = 0.45), with no evidence that any priming treatment compromised survival during the experiment (Figure S1 and Appendix S1). Further, to assess the absence of detectable adverse effects of priming in natural and aquaculture contexts, a second batch of H‐, K‐, and M‐primed clams was seeded in the Venice Lagoon during summer. Temperature records showed that these field‐exposed clams experienced two temperature peaks (>32°C) for short periods, while no heat waves were recorded (Figure S2 and Appendix S1). Since all groups experienced limited thermal stress, it is not surprising that no significant differences in CI (*p* = 0.311) and mortality emerged among treatments by the end of the summer (Table S1 and Appendix S1). Importantly, the absence of treatment‐specific losses in the natural environment demonstrates that chemical priming does not impair survival or performance when clams are returned to their habitat. Overall, results from both laboratory and field assessments support the physiological safety of priming treatments under the conditions tested. However, potential trade‐offs in traits not assessed here or over longer time scales cannot be excluded. Notably, although condition index did not change, other endpoints such as burrowing behavior and transcriptomic profiles indicate that the controlled heatwave induced a measurable, sub‐lethal stress response (see Results below).

### 3.3 Chemical Priming Improved Burrowing Capability During Heatwave Exposure

We then used burrowing behavior as an additional proxy to monitor priming effects at physiological and phenotypic levels. Indeed, this represents a well‐established metric for assessing clam physiological status, as it is known to be affected by environmental stressors [44, 45]. Beyond its physiological relevance, burrowing behavior may buffer clams against thermal extremes [46, 47], potentially reducing mortality risk. Furthermore, rapid burrowing also has an important ecological function: indeed, by allowing clams to quickly retreat below the sediment surface, it reduces their exposure to predators and can therefore lower mortality risk.

**FIGURE 2:**
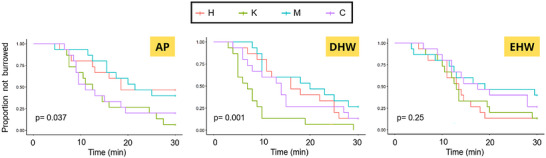
Burrowing kinetics following priming and MHW. Kaplan‐Meier survival curves show the probability of animals not having burrowed over time. The event is complete burrowing. Animals were tested at four time points: after priming (AP), after 7 days of MHW (DHW), and at the end of the heatwave (EHW). Log‐rank test p‐values are reported.

**FIGURE 3:**
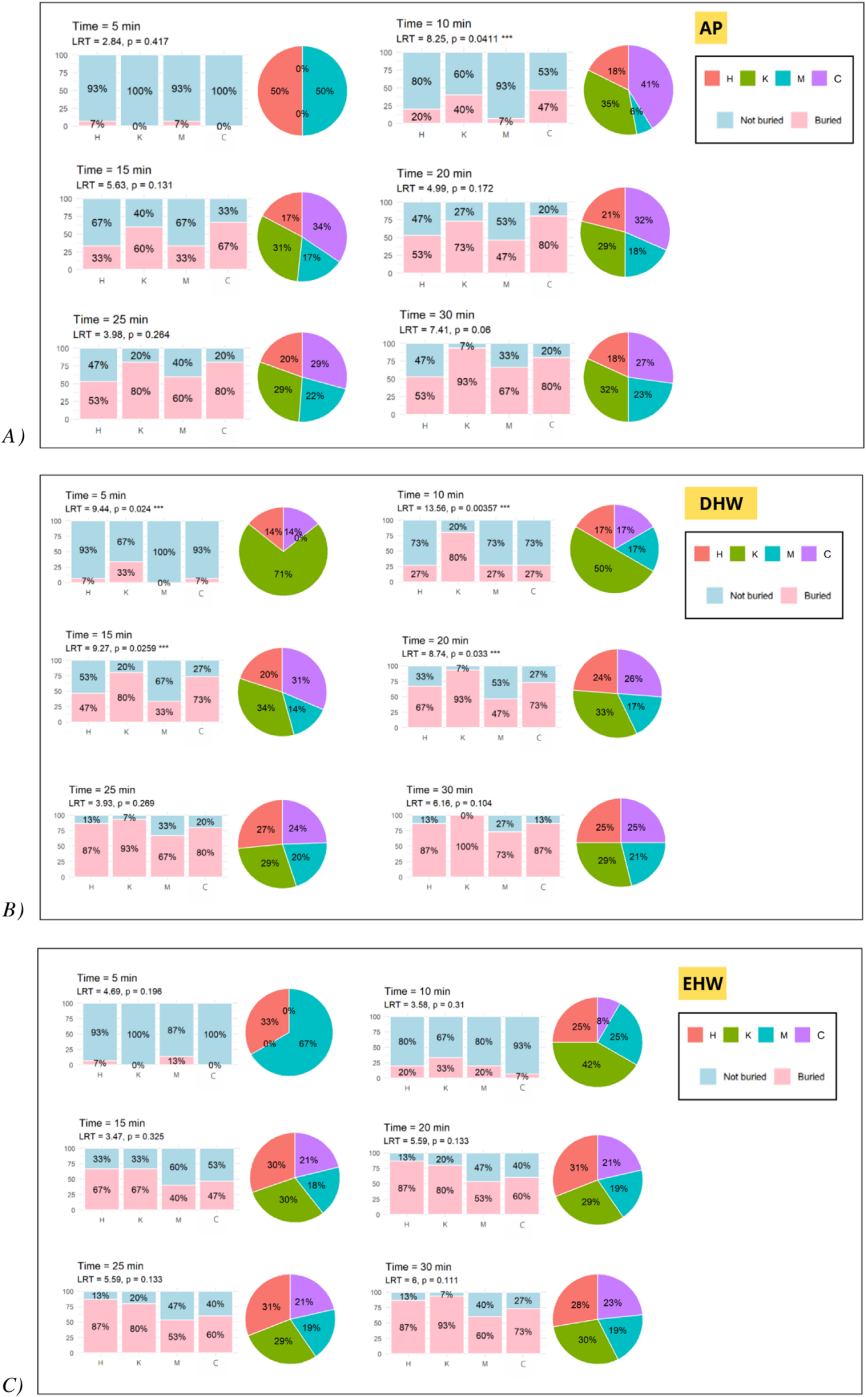
Burrowing Kinetics after priming (AP; A), during heat wave (DHW; B) and at the end of heat wave (EHW; C) detected after 5, 10, 15, 20, 25, and 30 min. Pie‐Charts show the proportion of burrowed clams corresponding to each treatment at each sampling time. Histograms show the percentage of burrowing events relative to each group.

Burrowing curves and burrowing kinetics are illustrated in Figures 2 and 3, respectively. Burrowing curves differed significantly after priming (log‐rank *p* = 0.037) and during the heatwave (log‐rank *p* = 0.001). After priming, both groups previously exposed to thermal priming (i.e., H and M) showed a markedly reduced burrowing performance compared with the control and chemically primed clams (Figures 2 and 3A). Such evidence is consistent with what was reported in ref. [22], where Manila clam burrowing time was significantly lower in thermally primed individuals compared with unprimed clams immediately following priming. In our experiment, this pattern was further supported by the Cox model, which indicated a slower burrowing tendency in H‐ and M‐primed clams (HR = 0.447 and 0.461; *p* = 0.079 and 0.081, respectively). The difference was particularly evident at 10 min (p‐value< 0.05), with a similar proportion of burrowed clams in the K and C groups (40% and 47%, respectively), whereas H‐ and M‐primed clams exhibited markedly lower burrowing proportions (20% and 7%, respectively). However, by 15 min the four groups converged toward similar burrowing proportions, reflecting differences in burrowing rates rather than in final burrowing capacity: thermal priming increased burrowing time without preventing successful burial, whereas chemical priming did not impose detectable constraints on burrowing performance.

During heatwave exposure, chemically primed clams (K) showed a significantly higher burrowing rate than controls (HR = 2.556; Cox p = 0.015), and overall, the best performance when compared to the other priming strategies (Figures 2 and 3B). At this stage, clear differences emerged among treatments. After 5 min, 71% of all burrowed clams belonged to group K, which had 33% of its individuals already buried, compared with only 0%–7% in the other groups, highlighting a markedly faster response (Figure 3B). At 10 min, group K continued to dominate the burrowing pool (50% of all burrowed clams), with 80% of its individuals already buried. In contrast, H, M, and C each contributed with 17% to the total number of burrowed clams and showed substantially lower burrowing proportions (27%). Despite such differences becoming less pronounced after 20 min, the K group remained statistically significant compared to controls. Notably, at 30 min, K was the only group in which all individuals were fully burrowed (Figure 3B).

At the end of the heatwave (EHW), neither burrowing curves nor burrowing dynamics differed significantly among treatments, although there seems to be a general trend indicating a better performance of K‐ and H‐primed clams, which tended to bury faster between 10 and 20 min. At 10 min, the K group showed the highest proportion of burrowed clams (42%), followed by H and M groups (25%), and the controls (6%) (Figure 3C). At 15 min, H and K groups displayed a higher percentage of buried clams (30%) than M and C groups (21%). By 20 min, burial reached 80% within K‐primed clams and 87% within H‐primed ones, while for controls (C) and mixed‐primed clams (M), the percentages were 60% and 53%, respectively. At later time points (25–30 min), differences among treatments persisted, with H‐ and K‐primed clams maintaining higher burial proportions compared to controls and mixed‐primed individuals. Although not statistically significant, these patterns suggest that priming effects may extend beyond burrowing rate to influence final burial outcomes (Figure 3C).

Taken together, these results indicate that priming treatments have distinct and time‐dependent effects on burrowing performance. Thermal priming, alone or in combination with chemical treatment, was associated with reduced burrowing performance immediately after priming, suggesting an initial physiological cost. In contrast, chemical priming did not impair burrowing and was associated with improved performance during heatwave exposure. Notably, the mixed treatment showed limited recovery across time points, with persistently lower burrowing performance, suggesting a cumulative physiological cost. Although not statistically significant, both H and K treatments tended to show improved performance at the end of the heatwave compared to controls, indicating a potential advantage under prolonged exposure.

### 3.4 Chemical Priming Induces Restricted and Distinct Transcriptomic Response Among Treatments

The analysis of transcriptomes is a well‐established method to understand an animal's physiology and health status, as transcript signatures can effectively assess complex gene expression responses to environmental conditions [48, 49, 50]. In this study, transcriptomic analyses were performed on digestive gland tissue, a key organ involved in metabolism, detoxification, and physiological regulation in bivalves. While this tissue is particularly informative for assessing stress‐related responses, it may not fully capture organism‐wide responses to priming and heat stress.

**FIGURE 4:**
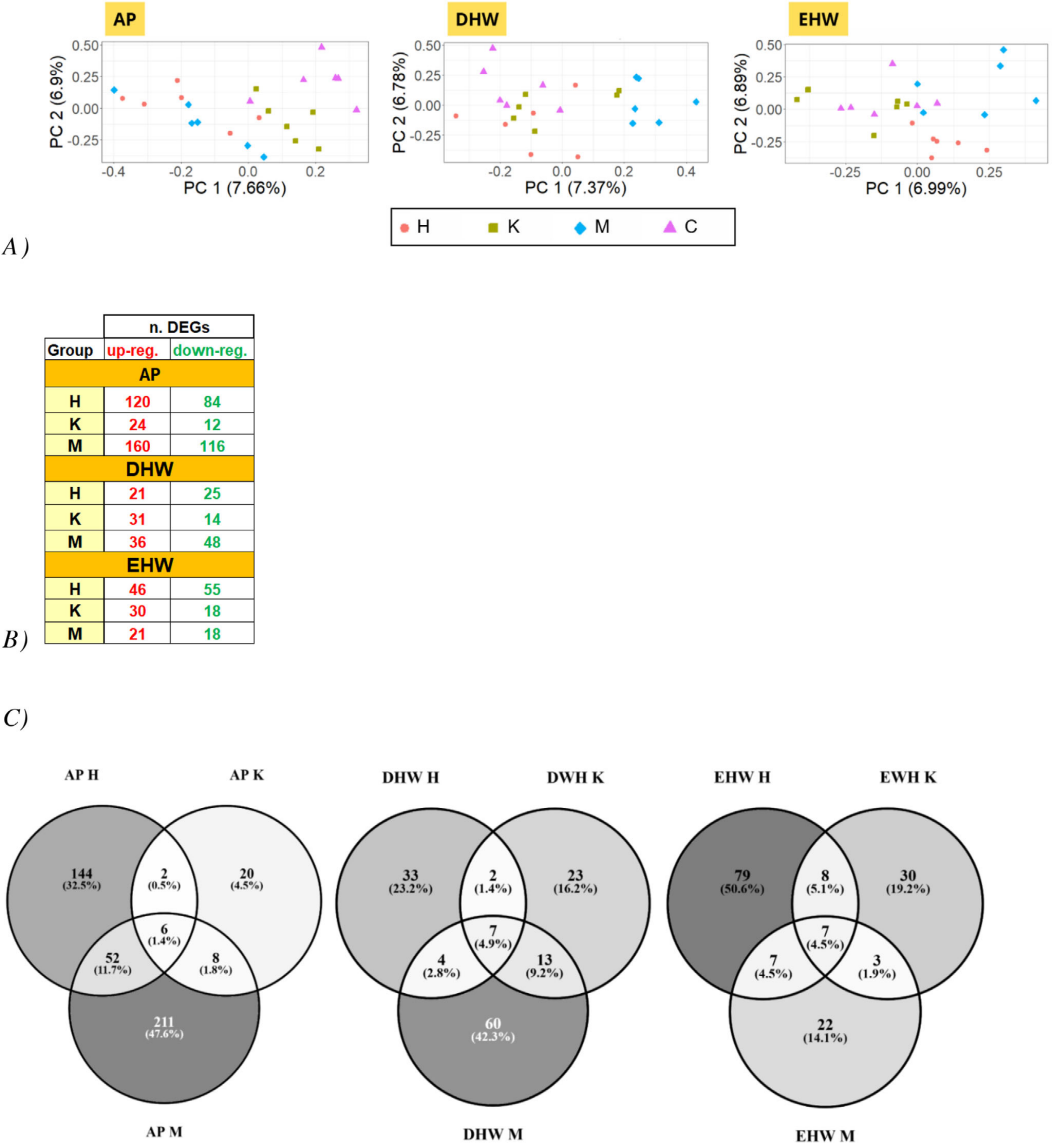
(A) PCA performed after priming (AP), during heat wave (DHW), and at the end of heat wave (EHW); (B) The table provides the number of differentially expressed genes (DEGs) for each treatment when compared to control group at corresponding sampling time (AP = after priming; DHW = during heat wave; EHW = end heat wave); (C) Venn diagrams report common DEGs revealed among treatments at each sampling time.

Principal component analysis revealed distinct clustering patterns across phases (Figure 4A). After priming (AP), H and M separated clearly from the control group (C), whereas K clustered closer to the control group (C), a pattern that was also observed during heatwave exposure (DHW and EHW). To formally assess the effect of treatment on global gene expression profiles, PERMANOVA analyses were performed separately at each time point. Treatment had a significant effect at AP (R^2^ = 0.150, *p* = 0.005) and EHW (R^2^ = 0.143, *p* = 0.029), while no significant effect was detected during heatwave exposure (DHW; R^2^ = 0.136, *p* = 0.078). To assess the extent of transcriptional changes induced by the heatwave, we also examined temporal variation in control samples. PCA revealed a clear trajectory across time points (AP, DHW, EHW), indicating that gene expression profiles changed over the course of the experiment in response to thermal exposure. This suggests that the imposed heatwave elicited a measurable transcriptional response (Appendix S1 and Figure S10). These observations were further supported by PERMANOVA analysis, which confirmed a significant effect of time on gene expression profiles in control samples (R^2^ = 0.20, *p* = 0.001). Additionally, pairwise comparisons showed that all three time points differed significantly from each other: AP vs. DHW (R^2^ = 0.15, *p* = 0.002), DHW vs. EHW (R^2^ = 0.12, *p* = 0.014), and AP vs. EHW (R^2^ = 0.20, *p* = 0.003).

The number of differentially expressed genes varied markedly across treatments and time points (Figure 4B). For the full list of significant DEGs and Gene Sets, see Appendix S2 and Appendix S3, respectively. The effects of priming and priming modes on the clam transcriptome mirror what was observed at the organismal level with the burrowing test. The strongest effect is shown by groups H and M, with the latter showing the highest number of DEGs compared to controls. In contrast, chemical priming (group K) is associated with limited changes in the clam transcriptome profile, with only 24 up‐regulated and 12 down‐regulated DEGs.

Venn diagrams revealed limited overlap in DEGs at all time points (Figure 4C), indicating that priming differentially shapes the clam transcriptome. However, genome‐wide overlap analysis revealed that M and H priming shared a significantly higher number of DEGs than expected (Fisher's exact test, *p* < 10^−40^), with a markedly stronger overlap than comparisons involving chemical priming (K). These results indicate that mixed priming elicits a transcriptional response more closely resembling thermal priming than chemical priming alone (Figure 4B,C). During and at the end of heatwave exposure, the overlap of DEGs among treatments remained limited and did not differ significantly across pairwise comparisons.

Functional analysis (GSEA) of differential expression, as a direct effect of thermal priming, revealed a clear positive enrichment of pathways associated with the response to thermal stress, including proteostasis and the unfolded protein response (e.g., protein folding, chaperone‐mediated protein folding, protein folding in the endoplasmic reticulum, and positive regulation of the ER unfolded protein response), together with transcriptional modulation of chaperones and heat shock proteins (HSPs). Consistently, pathways related to proteasome activity and ER‐associated degradation (*ubiquitin‐dependent ERAD pathway)* and to the response to ER stress (*intrinsic apoptotic signaling pathway in response to ER stress, response to endoplasmic reticulum stress*) were also significantly up‐regulated, involving ubiquitin‐conjugating enzymes and E3 ligases, indicative of enhanced removal of misfolded or thermally damaged proteins (Appendix S2 and Appendix S3). Additionally, enriched categories encompassed stress‐responsive signaling, including *response to oxidative stress, regulation of the stress‐activated MAPK cascade*, and *regulation of the JNK cascade*, pathways that mediate cell survival and proliferation, inflammatory‐like responses, and autophagy. Up‐regulation of MAPK/JNK signaling may also contribute to the modulation of cell cycle regulation (*regulation of cell cycle process; mitotic cell cycle process*), apoptosis (*execution phase of apoptosis*), and immune‐related responses (e.g., *regulation of immune response*), as suggested by other studies [51, 52]. Gene sets associated with lipid and sterol metabolism and membrane remodeling (e.g., *glycolipid metabolic process; sphingolipid metabolism*) were also enriched, reflecting the need to stabilize membranes perturbed by thermal stress [53, 54, 55]. Furthermore, consistent with the documented involvement of neurotransmitter signaling in bivalve stress physiology (Kotsyuba and Dyachuk, 2023) [56], we observed enrichment of gene sets related to behavioral processes (e.g., behavior, learning, memory). However, this result should be interpreted cautiously, as functional annotations of behavior‐associated genes are largely derived from vertebrate evidence and may not fully translate to distant taxonomic groups, unlike highly conserved cellular stress‐response pathways. Overall, the observed transcriptional signatures indicate a negative impact at the cellular level of thermal priming. This scenario is consistent with the significant reduction in burrowing performance observed in heat‐primed clams.

Chemical priming elicited a limited immediate response, with just 36 DEGs. GSEA identified only three significantly enriched pathways, including *ribosome* and *cytoplasmic translation*, both down‐regulated. These signatures point toward a generalized suppression of protein synthesis, which could be interpreted as a shift toward energy conservation and reduced metabolic expenditure without activation of classical stress pathways. In fact, chemical priming did not induce unfolded protein response, ER‐stress markers, MAPK/JNK signaling, or apoptosis‐ and immune‐related pathways, in sharp contrast with the broad stress program activated by thermal priming. Beyond translational suppression, chemical priming appears to induce a more selective, redox‐centered transcriptional program, characterized by the repression of secretory and barrier‐related genes, such as *mucin‐2‐like* and *WAP‐type four‐disulfide core proteins*, and the activation of genes involved in peroxisomal β‐oxidation, including *acyl‐CoA oxidase 1 (ACOX1), acetyl‐CoA acetyltransferase (ACAT*), and *3‐ketoacyl‐CoA thiolase B (ACAA1B*). Peroxisomal β‐oxidation, besides generating reactive oxygen species (ROS) as metabolic by‐products, determines an increase in lipid turnover, which plays a crucial adaptive role in bivalves, supporting membrane integrity under changing environmental conditions [57, 58]. Additional differentially expressed genes, including the up‐regulation of *SAMHD1‐like*, involved in nucleotide and redox homeostasis [59], and *protocadherin Fat1‐like*, together with the down‐regulation of extracellular matrix‐ and guidance‐related genes such as *neurocan‐like* and *semaphorin‐5A‐like*, suggest a subtle modulation of cell adhesion and extracellular matrix organization rather than a strong stress‐induced remodeling. Overall, chemical priming generated a low‐cost, non‐stressful transcriptional shift characterized by reduced protein synthesis, activation of peroxisomal lipid metabolism, and modest structural and regulatory adjustments. While these metabolic and translational changes have also been reported after thermal priming treatments [22], thermal priming elicits a much broader and energetically demanding stress response. This profile is fully consistent with the absence of behavioral impairment after priming and with the superior burrowing performance observed during heatwave exposure, indicating that chemical priming preserves physiological functionality while preconditioning cellular metabolism in a manner that may enhance thermal resilience.

Mixed priming (M) elicited the largest transcriptional effects, with a profile that significantly overlapped with the thermal priming response (Figure 4). This pattern may be consistent with the reduced persistence of H_2_O_2_ under elevated temperature conditions (see above), potentially resulting in a transcriptional response more similar to thermal priming alone. Consistently, several canonical heat‐stress components were up‐regulated, including *Heat shock protein 70 B2, protein disulfide‐isomerase A5‐like isoform X1 (PDIA5), Serine/threonine‐protein kinase/endoribonuclease ire‐1 (IRE1)*, and multiple ubiquitin‐ and proteasome‐associated genes, supporting the activation of proteostasis, ER stress, and unfolded‐protein‐response pathways highlighted also by GSEA (e.g. *Protein processing in ER, ERAD pathway, response to endoplasmic reticulum stress*). GSEA pointed out the up‐regulation of several immune‐related categories (e.g. *immune response, response to cytokine*), cytoskeletal and morphogenetic processes (*actin cytoskeleton organization, cell–cell adhesion*), and extensive signaling activity, including *MAPK cascade, NF‐κB signaling*, and synaptic signaling (e.g. *neuron projection development, synaptic signaling*). Moreover, several detoxification components were up‐regulated, including several ABC transporters (*ABCA5‐like*), *glutathione S‐transferase Mu 1‐like* (*GST‐Mu‐1‐like*), and *cytochrome P450*, pointing to an upregulated xenobiotic‐metabolizing system. Mixed priming also shared a subset of transcriptional changes with chemical priming, including *Ecdysone‐inducible protein E75*. E75 is a heme‐binding nuclear receptor whose activity in protostomes is tightly regulated by the intracellular redox environment [60, 61]. The shared up‐regulation of E75 in both K and M groups suggests oxidative signaling triggered by H_2_O_2_. Likewise, down‐regulation of the same gene sets identified in H_2_O_2_‐primed clams (*ribosome*, *cytoplasmic translation*, and *proteasome*) was observed in combined priming, suggesting partial suppression of protein synthesis. Overall, mixed priming triggered the widest and putatively most energetically demanding transcriptional program, characterized by simultaneous activation of ER‐stress, immune, cytoskeletal, detoxification, and oxidative stress signaling pathways. All such evidence points toward a major impact at the cellular level, likely due to the cumulative effect of chemical and thermal stress. This hypothesis is consistent with the burrowing test results at the AP time point, where the M group showed the poorest performance. Such a cumulative effect appears to have long‐term consequences, as burrowing behavior remained negatively affected after one and two weeks of HW challenge.

Upon thermal challenge, subtle deviations from the heat stress response of control clams were observed in all three primed groups. Because differential expression was assessed relative to heatwave‐exposed controls, transcriptional changes detected during and after the heatwave reflect priming‐dependent modulation of the baseline stress response. Within this framework, H, K, and M treatments displayed distinct yet partially overlapping transcriptional signatures, indicating that priming history shapes heat‐stress regulation in a treatment‐specific manner.

After one week, only a small number of DEGs were detected, and no significant GSEA enrichment was observed. Heat‐primed clams exhibited down‐regulation of several transcriptional regulators, including *LIM homeobox 1‐beta*, an RNA polymerase subunit, and *Signal transducer and activator of transcription* (*STAT*). In contrast, *tubulin β‐4B* and *fibrillin‐2*, both associated with structural roles in tissue integrity (Tuszynski et al. [62], 2006; Ramirez and Sakai, 2010) [63], were up‐regulated, together with the thermosensitive *transient receptor potential cation channel subfamily A member 1* (*TrpA1*), a key component of heat‐response pathways in marine invertebrates (Peng et al., 2021; [64] Zhang et al., 2025, [65]. Notably, *heat shock 70 kDa protein 12B‐like* (*HSPA12B*) was up‐regulated in K‐primed clams during heatwave exposure, whereas H‐ and M‐primed clams showed down‐regulation of *HSPA12A‐like* and *HSPA12B*. This priming‐specific pattern suggests that chemical priming modulates the heat stress response through distinct molecular components, preserving the inducibility of canonical heat‐shock proteins rather than pre‐activating them during the priming phase, as observed in H‐ and M‐primed clams.

The chemical‐primed group (K) showed down‐regulation of *hexokinase‐1*, a glycolysis‐related gene known to decrease under heat stress [66], while displaying up‐regulation of *fibrillin‐2* (commonly to H) and *big defensin*, which plays crucial roles in clams’ immune function thanks to its antimicrobial activity [67, 68]. Among the genes exclusively shared between K and M, we identified the *E75‐like nuclear hormone receptor* up‐regulated in both treatments also after priming. The continuous up‐regulation of *E75* in K‐ and M clams reflects a clear transcriptional sensitivity to H_2_O_2_. Given the potential role of *E75* orthologs in oxidative‐stress signaling and cellular remodeling in protostomes [69, 70], its activation during the heatwave suggests that oxidative‐priming‐responsive pathways remained active under thermal stress.

At the end of the heatwave, H‐primed clams displayed differential expression of genes associated with extracellular matrix and cell‐surface organization (e.g., *mucin‐like proteins, cadherin/protocadherin family members, papilin‐like*), immune and detoxification processes (*complement factor B‐like, galectin‐related protein, cytochrome P450 10‐like, heavy metal‐binding proteins*) and cell‐cycle and proteostasis regulation (*cyclin‐B3, DNA polymerase subunit 4, DNA replication complex GINS protein SLD5*, several E3 ubiquitin‐protein ligases). In contrast, K‐ and M‐primed clams exhibited fewer differentially expressed genes relative to naïve controls (C), without consistent pathway‐level enrichment. Notably, a shared transcriptional feature across all primed groups (H, K, and M) was the up‐regulation of the *Death‐associated inhibitor of apoptosis 2* (*DIAP2*). *DIAP2* is a conserved inhibitor of apoptosis protein that limits caspase‐mediated cell death, promoting cell survival under stress conditions [71, 72]. Its consistent up‐regulation across all primed groups at the end of the heatwave suggests that priming, irrespective of its mode, promotes the engagement of anti‐apoptotic mechanisms during prolonged thermal stress.

Taken together, these results indicate that, compared to thermal priming, chemical priming was associated with limited transcriptional perturbation while preserving physiological performance as suggested by burrowing capability, suggesting a distinct mechanism for modulating thermal resilience under simulated heatwave conditions.

### 3.5 Microbiota Characterization Reveals Limited Community Shifts and Reduced Representation of Potentially Opportunistic Taxa in Primed Clams During Heatwave Exposure

Microbiota is a key determinant of host health, contributing to physiological homeostasis, stress resilience, and detoxification processes [73, 74]. Environmental stress and marine heatwaves can disrupt microbial communities, impairing their capacity to support the holobiont [4, 75, 76]. Hydrogen peroxide is known to exert antimicrobial effects at sufficiently high concentrations, while at lower doses it primarily acts as a redox‐active signaling molecule. Although potential bactericidal effects at the concentrations applied here cannot be assumed, chemical priming could nonetheless influence host‐microbiota interactions and thereby reshape microbiota composition or dynamics. Moreover, previous work in *Ruditapes philippinarum* has shown that thermal priming can alter microbial community structure and suggests that priming history may influence microbiota dynamics following heatwave exposure [22]. For these reasons, the bacterial communities associated with the clams were profiled to determine whether priming induced specific microbiota shifts or imbalances relative to non‐primed controls.

**FIGURE 5:**
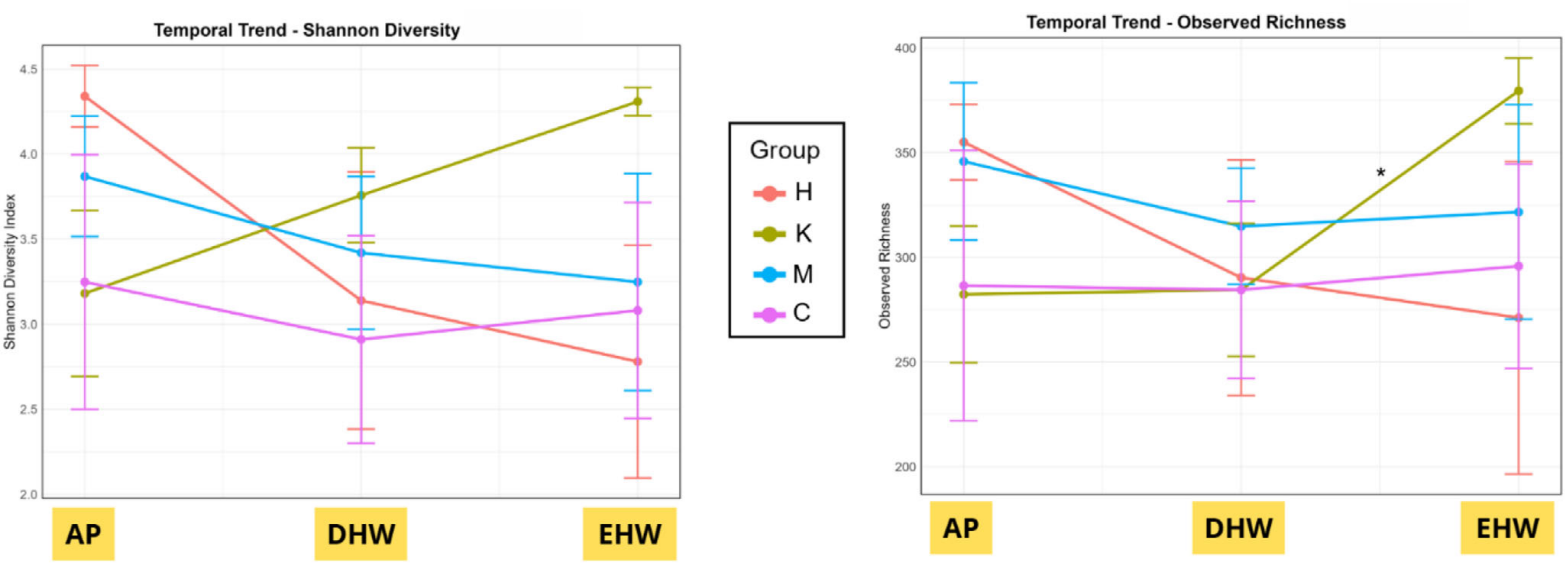
Shannon diversity (left) and Observed Richness (right) indices across phases (AP = after priming, DHW = during the heatwave, EHW = at the end of the heatwave) among different treatment groups of clams (H = heat priming, K = chemical priming, M = mixed priming, C = control). Asterisks indicate the significant temporal contrast (Wilcoxon rank‐sum test).

Alpha diversity did not differ significantly among groups within each sampling phase (Figure S3 and Appendix S1). However, temporal trends revealed treatment‐specific trajectories (Figure 5). Both H and M displayed an initial increase in diversity immediately after priming compared to the non‐primed C group, followed by a decline at DHW and stabilization toward EHW. In contrast, K exhibited the opposite temporal profile, with diversity increasing progressively from AP to DHW and EHW. This trend was supported by a significant global temporal effect in the Observed Richness index (Kruskal‐Wallis *p* = 0.0393), driven by a significant rise between DHW and EHW (Wilcoxon adjusted *p* = 0.0391). The control group (C) remained comparatively stable across phases. Overall, despite high inter‐individual variability (Figure 5), alpha‐diversity analyses indicate that priming did not induce persistent alterations in within‐sample diversity but was associated with transient, treatment‐specific temporal patterns.

Beta‐diversity analyses showed homogeneous dispersion across groups in all experimental phases, supporting the validity of PERMANOVA analysis. Across all time points, PCoA showed no clear clustering of samples by treatment, indicating that priming did not induce strong, visually detectable restructuring of the microbiota (Figure S4 and Appendix S1). Statistical tests (PERMANOVA) performed on the same distance matrices revealed only modest and phase‐specific effects (Appendix S1). Together, these results indicate that neither thermal nor chemical priming produced major community‐wide shifts in microbiota composition, and that any statistically detectable differences were limited in magnitude and context‐dependent.

**FIGURE 6:**
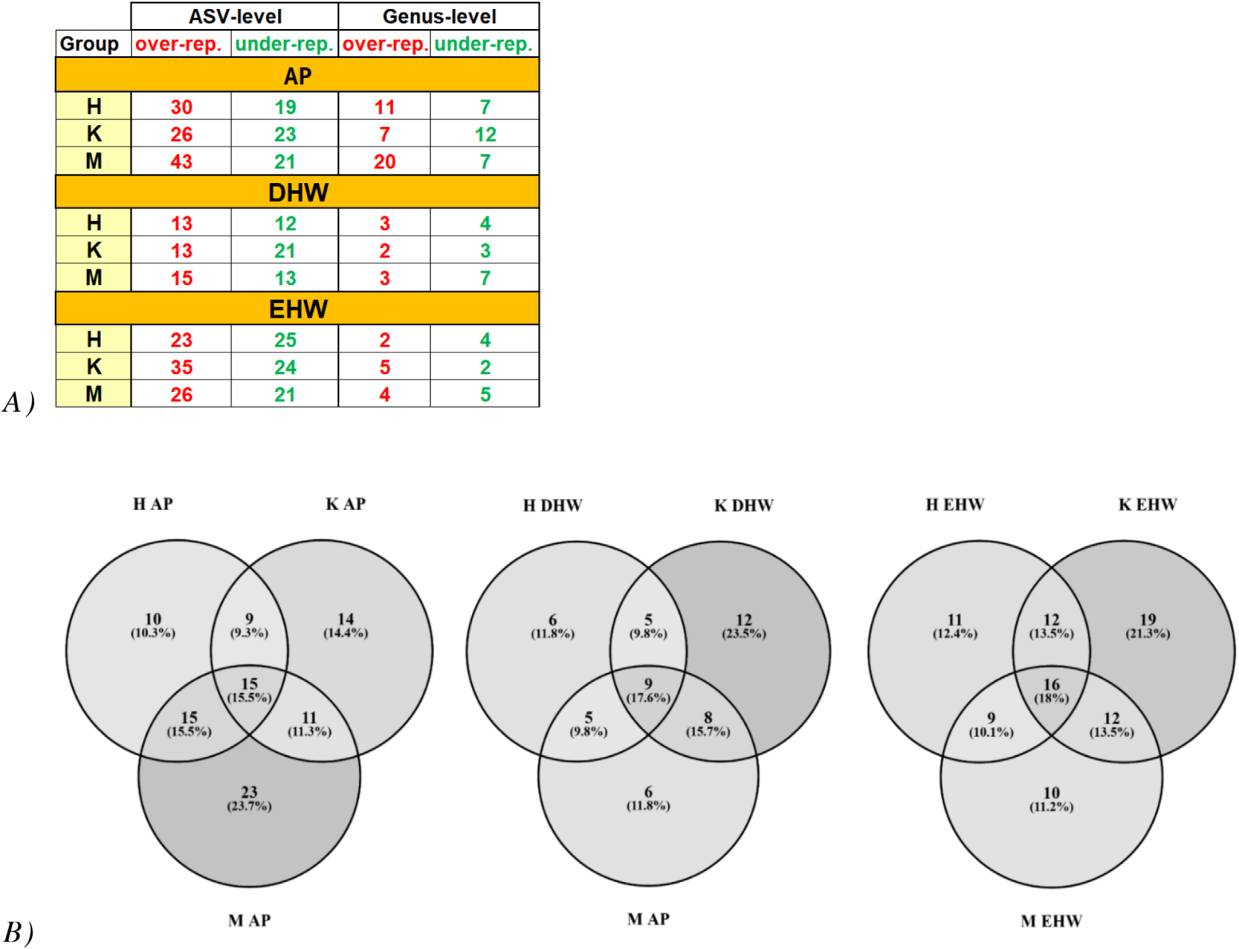
(A) Table with differential abundant taxa both at ASV and genus level in the clam digestive glands, obtained comparing group H (heat priming), K (chemical priming), and M (mixed priming) with group C (control), at each experimental phase: after priming (AP), during the heatwave (DHW), and at the end of the heatwave (EHW). (B) Venn diagrams showing the number and proportion of differentially abundant ASVs uniquely or jointly detected among primed groups at each experimental phase (made using Venny 2.1.0).

Across phases, the number of differentially abundant ASVs in each treatment compared to the C group varied markedly (Figure 6). Differential abundance analyses (FDR‐corrected) identified phase‐specific changes in the relative abundance of bacterial taxa across priming treatments (Appendix S4). During priming (AP), M showed the highest number of differentially abundant taxa (DAT), while at the end of the heatwave (EHW), the number of DAT increased compared to DHW in all treatments, especially in K, consistent with the increased index of alpha diversity.

After priming (AP), all primed groups showed significant enrichment of *Vibrio*, including *V. mediterranei* and *Shewanella algae*, two species often linked to dysbiosis, stress responses, or pathogenicity in bivalves [77, 78]. However, the magnitude of this shift differed across treatments. Specifically, relative to control clams, H‐ and M‐primed clams showed enrichment of multiple *Vibrio* amplicon sequence variants (ASVs) (four and five ASVs, respectively), indicating a broader expansion within the *Vibrio* lineage, whereas K‐primed clams displayed enrichment of only a single *Vibrio* ASV. In addition, the M‐primed group uniquely exhibited higher abundance of *Photobacterium*, a genus that includes known bivalve pathogens [79]. Among these opportunistic pathogens, *Vibrio* remained consistently enriched across all groups during the heatwave. Notably, by the end of the heatwave (EHW), several ASVs belonging to *Vibrio* and *Tenacibaculum* were significantly under‐represented in all primed groups relative to controls. *Tenacibaculum* species have been implicated in shellfish diseases, including lesions and mortality in clams and oysters [80, 81]. *Mycoplasma*, a dominant and potentially beneficial genus in *R. philippinarum* [82], exhibited clear temporal shifts. By the end of the heatwave, it was strongly over‐represented in K and M and moderately increased in H‐primed clams, while remaining comparatively lower in the control group (C). Although *Mycoplasma* is often associated with stable clam microbiota [82, 83], the functional implications of its increased relative abundance under thermal stress remain incompletely understood and should therefore be interpreted cautiously.

Overall, these results indicate that priming elicited transient and treatment‐specific changes in microbiota composition, particularly in M‐ and H‐primed clams after priming, which largely diminished during heatwave exposure. Notably, taxa commonly associated with dysbiosis (e.g., *Vibrio, Photobacterium, Tenacibaculum*) were over‐represented in primed groups prior to the heatwave but showed reduced relative abundance during or after thermal exposure in primed clams compared to non‐primed controls. Together with modest shifts in beta‐diversity and stabilized alpha‐diversity patterns, these results indicate that priming does not cause persistent microbiota disruption. Instead, primed individuals exhibit microbiota dynamics characterized by reduced persistence of potentially opportunistic taxa following thermal stress compared to non‐primed controls.

### 3.6 Mechanistic Insights and Broader Implications

Our study provides the first evidence that low‐dose hydrogen peroxide chemical priming modulates organismal responses to thermal stress. The experimental heatwave induced measurable responses in control clams, particularly at the behavioral and transcriptomic levels, indicating a moderate, sub‐lethal stress condition. This reveals a mechanism by which mild oxidative cues pre‐condition organismal and holobiont responses to simulated heatwave conditions. Unlike thermal priming, which was associated with broader transcriptional activation and transient behavioral impairment, chemical priming was associated with limited transcriptomic perturbation, preserving physiological performance while still modulating stress‐responsive molecular components during heat exposure. Chemically primed clams showed faster burrowing rates during heatwave exposure and displayed distinct transcriptional profiles, while microbiota communities exhibited transient and treatment‐specific shifts, including reduced persistence of opportunistic taxa such as *Vibrio* and *Tenacibaculum* during and after heat stress compared to non‐primed clams. Finally, a field trial conducted under commercial farming conditions confirmed the safety of chemical priming. From an applied perspective, the simplicity and feasibility of H_2_O_2_ priming may support the development of low‐impact approaches to improve bivalve performance under warming conditions. Although tested here in Manila clams, this redox‐mediated priming mechanism may provide a broader framework for modulating stress memory across sessile marine invertebrates under moderate thermal stress scenarios. Future studies should explore its applicability under more intense or prolonged marine heatwave scenarios, and across taxa with differing life histories and thermal tolerances.

## Author Contributions

Massimo Milan and Maura Benedetti secured funding for this study. Massimo Milan, Luca Peruzza, Chiara Mascitelli, and Giovanna Monticelli coordinated the overall experimental design and execution. Chiara Mascitelli, Giovanna Monticelli, Ilaria Bernardini, and Giulia Dalla Rovere performed clam priming, simulated heatwave exposures, burrowing assays, and measurements of survival and condition index, and conducted the field experiment. Chiara Mascitelli carried out the molecular analyses, including wet‐lab procedures and bioinformatic analyses. Graziano Rilievo quantified H_2_O_2_ decay in experimental seawater. Luca Peruzza and Giulia Dalla Rovere contributed to bioinformatic analyses. Alessandro Nardi, Deborah Cesaroni, and Valentina Tavolazzi contributed to clam sampling and supported experimental exposures. Chiara Mascitelli and Massimo Milan drafted the manuscript. Tomaso Patarnello, Gabriele Andreatta, Luca Peruzza, Giovanna Monticelli, and Luca Bargelloni contributed to experimental planning and manuscript revision. All authors contributed to the final version of the manuscript and approved its submission.

## Conflicts of Interest

The authors declare no conflict of interest.

## Supporting information




**Supporting File 1**: gch270123‐sup‐0001‐SuppMat.docx.

**Supporting File 2**: gch270123‐sup‐0002‐AppendixS1.docx.

**Supporting File 3**: gch270123‐sup‐0003‐AppendixS2.xlsx.

**Supporting File 4**: gch270123‐sup‐0004‐AppendixS3.xlsx.

**Supporting File 5**: gch270123‐sup‐0005‐AppendixS4.xlsx.

## Data Availability

The data that support the findings of this study are openly available in NCBI at https://www.ncbi.nlm.nih.gov/, reference number PRJNA1391265.
