# Supplementary material for: Low‐Dose H2O2 Priming Improves Performance Under Simulated Marine Heatwave Conditions in a Coastal Bivalve
Source: Glob Chall. 2026 Jun 14;10(6):e70123. doi: 10.1002/gch2.70123 (PMC13266081; doi:10.1002/gch2.70123)
Supplement: Supplementary file 1 — Supporting File 1: gch270123‐sup‐0001‐SuppMat.docx. [file GCH2-10-e70123-s001.docx]

SUPPLEMENTARY APPENDIX A

MATERIALS AND METHODS

**Animals and experimental procedures**

Juvenile *Ruditapes philippinarum* (mean shell length ± SD: 20.13 ± 1.48 mm; mean wet mass ± SD: 3.75 ± 0.81 g) were purchased from SATMAR (France) in April 2025. Upon arrival, clams were held for 2 h in a 45-L aerated container for depuration and recovery. All experiments were conducted using artificial seawater prepared in the laboratory and adjusted to a salinity of 28.5-30.5 PSU.

Clams were maintained in 60-L two-compartment (“bi-chamber”) tanks: the upper chamber housed the animals, and the lower chamber contained a continuous-flow recirculation pump, mechanical (sponge) filtration, biological filtration media, and an air stone for aeration. Each tank held ~65 clams, and 30% of the water volume was replaced weekly with freshly prepared artificial seawater.

Temperature was continuously logged using iButton DS1921H-F5 sensors and controlled via 100 W submersible heaters connected to a precision thermoregulator (MPT91, Mect, Italy), programmed for constant or time-varying thermal profiles. Clams were maintained under a continuous light-dark (LD) cycle (LD14:10), and fed daily with New Coral Fito Concentrate (A.G.P., Italy), a microalgal blend composed of Isochrysis (T-Iso; 33.3%), Nannochloropsis (31%), Tetraselmis (18%), and Phaeodactylum (18%), administered at a rate of 1 mL per 15 clams diluted in tank water.

The priming treatment was repeated on a second batch of clams, which were transferred to the field immediately after the treatment. Prior to deployment, clams were measured (shell length, shell width, and total weight) and individually tagged by gluing plastic bee tags onto their shells using cyanoacrylate glue. They were then placed in three adjacent underwater cages in the Chioggia Lagoon (45.1932218 N, 12.2539639 E), within a farming site that experienced serious HW-related mortality events in the past. Clams were inserted into HDPE bivalve pouches with zips, mounted at the same height across the cages. Water temperature and mortality were continuously monitored using temperature loggers. At the end of the trial, clams were recovered and sampled for condition index determination. Differences in mortality among treatments were assessed using a chi-square test applied to contingency tables of dead versus surviving individuals pooled across cages.

**Burrowing test**

Burrowing behavior was assessed at three time points: after priming (AP), midway through the heatwave (DHW), and at the end of the heatwave (EHW). At each time point, a random subset of 15 clams per treatment (5 from each replicate) was tested. Trials were conducted in plastic containers filled with aerated seawater at 25 °C and 4 cm of sand, with groups separated by dividers. A small recirculation pump ensured gentle water mixing. A Logitech C920 PRO HD camera mounted overhead recorded continuous videos for 30 min. Latency to complete burial was defined as the time when the entire shell became fully covered by sediment.

Kaplan-Meier curves and log-rank tests were used to compare time-to-burial distributions among treatments. A Cox proportional hazards model quantified differences in burial rates (hazard ratios), using the non-primed control (C) as the reference group. Individuals not buried within 30 min were right-censored. At fixed time points (i.e. every 5 min up to 30 minutes), the proportion of buried clams was analyzed using binomial Generalized Linear Models (GLMs), with likelihood ratio tests used to compare models including or excluding treatment as a predictor.

**Transcriptomic analysis**

Digestive glands were dissected from clams at AP, DHW, and EHW. Tissues were immersed in RNAlater® (ThermoFisher, USA), held at -20 °C overnight, and stored at −20 °C. Total RNA was extracted using the RNeasy Mini Kit (QIAGEN) following manufacturer instructions. RNA concentration and purity were assessed with a Qubit Fluorometer (Invitrogen, USA), and integrity was confirmed on an Agilent 2100 Bioanalyzer, retaining only samples with concentration >20 ng/µL and RIN ≥ 7.

Library preparation and sequencing were performed by Biomarker Technologies (BMK, Germany) on an Illumina NovaSeq X platform (paired-end 150 bp ).

Raw reads were processed using nf-core/rnaseq (v3.14.0; Nextflow^1^ 25.04.7, Singularity profile). Transcript quantification was performed with RSEM, using the *Ruditapes philippinarum* reference genome (unpublished). Differential expression analysis used edgeR (v4.4.2) with RUVSeq^2^ (v1.40.0) for normalization (k=2 for AP; k=5 for DHW and EHW). Lowly expressed genes were filtered using filterByExpr. Differentially Expressed Genes (DEGs) were defined using the quasi-likelihood framework, with FDR ≤ 0.1 and |log₂FC| ≥ log₂(1.5).

Gene annotation relied on a custom Gene Transfer Format (GTF) annotation. Functional enrichment and Gene Set Enrichment Analysis (GSEA) were performed with clusterProfiler^3^ (v4.14.6) and DOSE^4^ (v4.0.1). GSEA used ranked gene lists with min/max gene set sizes of 5-500 and FDR ≤ 0.2.

**Microbiota analysis**

Total RNA extracted from digestive glands (the same samples used for transcriptomics) was reverse-transcribed to cDNA and used as template to amplify the V3-V4 region of the bacterial 16S rRNA gene. PCRs were performed using primers 338F (5′-ACTCCTACGGGAGGCAGCA-3′) and 806R (5′-GGACTACHVGGGTWTCTAAT-3′) with a high-fidelity polymerase under standard cycling conditions . Amplicon libraries were prepared and sequenced by Biomarker Technologies (BMK GmbH, Germany) on an Illumina NovaSeq platform to obtain paired-end 2×250 bp reads. Raw reads were processed using the nf-core/ampliseq pipeline (version 2.14.0; Nextflow^1^ 25.04.7, Singularity profile). The option --illumina_novaseq was applied, and truncation lengths were automatically selected based on quality scores (mean Q < 25). Outputs (ASV table, taxonomy, phylogenetic tree) were imported into phyloseq^5^ (v1.50.0) in R (v4.4.2). Taxa with <10 total reads or present in <3 samples were removed. Data were transformed into relative abundances for community-composition analyses. Alpha diversity (Shannon index and Observed Richness) was computed using estimate_richness () and compared among groups within each phase (AP, DHW, EHW) using Kruskal-Wallis tests. Temporal changes within each treatment were assessed with Wilcoxon rank-sum tests between consecutive phases, with p-value adjustment. Beta diversity was assessed using Bray-Curtis and weighted UniFrac distances. Principal Coordinates Analyses (PCoA) were generated for visualization. Statistical significance of community structure was tested via PERMANOVA (adonis function, vegan v2.7-1). Homogeneity of multivariate dispersion was checked using the betadisper function in the R package vegan^6^. When PERMANOVA models were globally significant, pairwise comparisons were performed, restricting contrasts to each priming group versus the non-primed control (H vs C, K vs C, M vs C). For taxonomic resolution at higher ranks, analyses were repeated after aggregating data at the genus level. Differentially abundant taxa (ASVs and genera) of each priming treatment compared with controls were identified using DESeq2^7^ (v1.46.0).

**H_2_O_2_** **Spectrophotometric assay**

The reagents used were: 4-amino-antipyrine (AMP, powder), N,N-dimethylaniline (DMA, 99%), horseradish peroxidase type II (HRP, 191 U/mg). All reagents were purchased from Sigma-Aldrich. Spectroscopy measurements were performed in 1 cm light path plastic cuvettes (Sarstedt) using a Cary 60 UV-visible spectrophotometer (Agilent Technologies). To collect the sample absorbance spectra, the following scan settings were applied: scan time 0.1 s, scan interval 1 nm, scan rate 600 nm min^-1^, wavelength range from 450 nm to 650 nm. A colorimetric assay to measure the hydrogen peroxide concentration in marine water was developed, inspired to the one proposed by ^8^. Briefly, H_2_O_2_ is used by HRP to catalyze the reaction between AMP and DMA, producing a colored compound which has a maximum absorption around 555 nm wavelength. An indirect quantification of hydrogen peroxide is therefore allowed. In detail, H_2_O_2_ concentration was monitored in the presence of 3 mM DMA, 4 mM AMP, and HRP 5 U mL^-1^. After adding the H_2_O_2_ to the reaction mix, the solution was mixed and incubated for 5 min. Then, the optical spectrum was collected. For quantification purposes, a calibration curve was built testing H_2_O_2_ standards in the 0-200 µM range. The marine water was previously tested in comparison to ultrapure water, in order to evaluate possible matrix effects for this assay. The assays were carried out at room temperature.

RESULTS

**Beta-diversity**

A significant effect of treatment condition on the overall microbial community structure was transient, appearing only in the early phases of the experiment, but its strength and detection varied by taxonomic resolution and phase.

At the Amplicon Sequence Variant (ASV)-level, significant treatment effect of treatment condition on the overall microbial community structure, as measured by Bray-Curtis dissimilarity, was observed exclusively during the AP phase (PERMANOVA: R² = 0.224, p = 0.002). In this phase, pairwise post-hoc tests with FDR correction identified the community in H treatment as significantly distinct from those in K (p-adj = 0.024), 'mixed' (M) (p-adj = 0.024), and control (C) (p-adj = 0.024) groups. However, analysis using the Weighted UniFrac distance metric in the same AP phase did not yield a significant result (R² = 0.192, p = 0.078 ). In the subsequent DHW phase, the overall Bray-Curtis model showed a marginal, non-significant trend (R² = 0.173, p = 0.067), while the Weighted UniFrac analysis confirmed no significant difference (R² = 0.157, p = 0.244). In the final EHW phase, no significant overall treatment effects wasere found (Bray-Curtis: R² = 0.135, p = 0.394; Weighted UniFrac: R² = 0.172, p = 0.221). Tests for homogeneity of multivariate dispersions (Betadisper) were non-significant for all phases and both distance metrics (all p > 0.05), confirming that the observed PERMANOVA results were not confounded by differences in group variances. In summary, a statistically significant divergence in microbial community composition driven by treatment was transient, detectable only in the initial AP phase using the Bray-Curtis dissimilarity index.

The statistical analysis was repeated after the agglomeration at the Genus genus level. A significant effect of treatment condition on the overall microbial community structure was observed in the AP phase for both distance metrics. The effect was significant for Bray-Curtis dissimilarity (PERMANOVA: R² = 0.262, p = 0.002) and for Weighted UniFrac (R² = 0.276, p = 0.001). Post-hoc tests for Bray-Curtis indicated the how H group was distinct from K (p-adj = 0.030), M (p-adj = 0.030), and C (p-adj = 0.045), while the K group also differed from C (p-adj = 0.030). For Weighted UniFrac, the H group differed from K (p-adj = 0.018) and M (p-adj = 0.018), and K differed from C (p-adj = 0.018). This significant pattern persisted into the DHW phase . Strong overall differences were again detected for both Bray-Curtis (R² = 0.276, p = 0.001) and Weighted UniFrac (R² = 0.254, p = 0.001). Pairwise comparisons for Bray-Curtis revealed several significant differences: H differed from K (p-adj = 0.031) and M (p-adj = 0.031); K differed from M (p-adj = 0.018) and C (p-adj = 0.031); and M differed from C (p-adj = 0.018). For Weighted UniFrac, significant pairwise differences were found between K and M (p-adj = 0.039) and between M and C (p-adj = 0.018). In the final EHW phase, the overall PERMANOVA model showed marginal, non-significant trends for both Bray-Curtis (R² = 0.162, p = 0.063) and Weighted UniFrac (R² = 0.175, p = 0.065). No specific pairwise comparisons remained statistically significant after FDR correction. The assumption of homogeneity of multivariate dispersions was met for all phases and both metrics (all Betadisper p > 0.05). In summary, after agglomeration to at the genus level, a strong and significant treatment effect of treatment on community composition was evident in both the AP and DHW phases, but was absent by in the EHW phase, indicating a transient, phase-dependent response.

**H_2_O_2_** **decay**

Firstly, the absorbance spectra resulting from H_2_O_2_ standard concentrations ranging from 0 µM to 200 µM in marine water were collected, following the colorimetric method previously described (**Figure S5**). That way, a calibration curve was made with the absorbance signal collected at 555 nm wavelength (**Figure S6**). The resulting H_2_O_2_ calibration curve gave an extinction coefficient ε of 5.05x10^-3^ µM^-1^ cm^-1^, used for the quantification of hydrogen peroxide in marine water with the developed colorimetric assay. After developing the quantification protocol, the evaluation of H_2_O_2_ concentration in the aquaria system during time was evaluated at room temperature.

First, the whole system without the presence of clams was assessed, as control measurement of the normal H_2_O_2_ decay in marine water. Hydrogen peroxide concentrations in aquaria were checked from its addition (*i.e.*, timepoint t 0 min) and for the next 24 h (**Figure S7**). As clearly visible in Fig.3, left panel, the compound degradation in marine water followed a first order decay kinetics, and its linearized form (**Figure S7**, right panel) gave a rate constant k of 0.19 ± 0.03 h^-1^. In practical terms, the presence of hydrogen peroxide in water was no longer detected after about 20 h.

Then, the H_2_O_2_ duration in the aquaria water was tested again in the presence of clams, at room temperature. In the same aquaria system, clams were put in acclimatation for 48 hours. After that, the hydrogen peroxide was added to water (t 0 min). Subsequently, a sample of marine water was collected at different timepoints, and the H_2_O_2_ levels were monitored (**Figure S8**). Again, a decrease in the hydrogen peroxide concentrations during time was observed, but with a faster rate compared to the controls with no clams (**Figure S8**, left panel). The system was in good accordance with a first order decay kinetics (**Figure S7**, right panel), and the linearized form of the respective equation gave a rate constant of k = 0.41 ± 0.08 h^-1^. That meant that the presence of H_2_O_2_ in those conditions was no longer detectable in acquaria with clams after 5-6 hours of exposition.

Due to this significative difference between the slow H_2_O_2_ decay in clean acquaria marine water and the faster decay rate in presence of clams, an effective interaction of the peroxide compound with the animals can be affirmed. In addition, the same decay test was carried out for the system with clams, but at 30°C temperature. Results can be seen in **Figure S9**. Also in this case, the decay followed the first order kinetics (**Figure S9**, left panel), but in this case in a slightly faster way compared to the system at room temperature, as predictable. Indeed, it is well affirmed that a rising in the environment temperature normally lead to a faster degradation rate of a compound. Therefore, the kinetic constant calculated here was of 0.80 ± 0.05 h^-1^ (**Figure S9**, right panel), two times greater than the one of the room temperature decay test.

SUPPLEMENTARY FIGURES


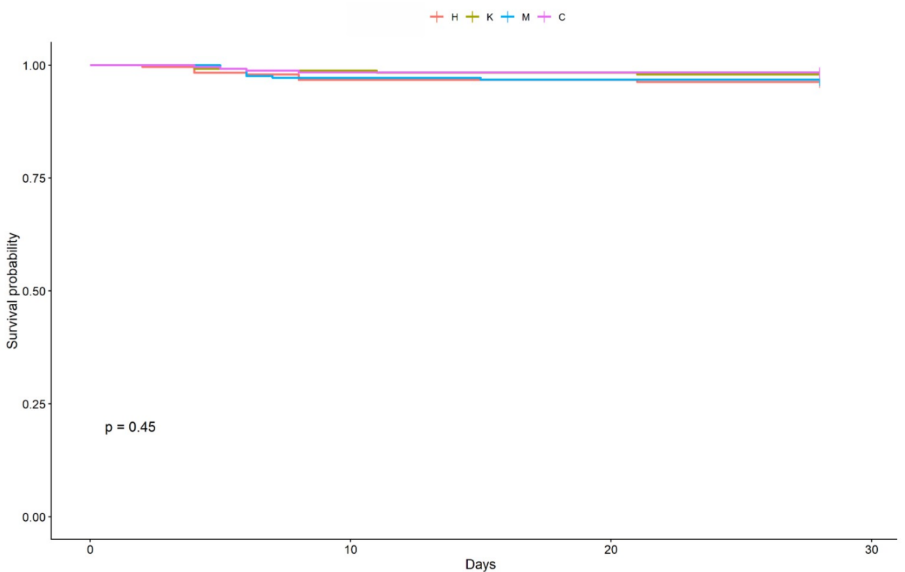


**Figure S1**: Kaplan-Meyer curves of mortality observed during the priming experiment among groups. Log-rank p-value is reported. H= heat priming, K= chemical priming, M= mixed priming, C= control.

**
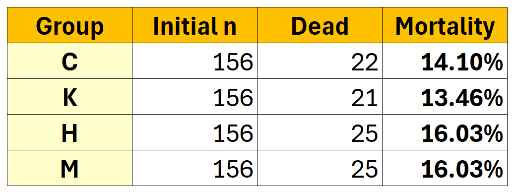
**

**Table S1**: Mortality expressed as the percentage of individuals lost relative to the initial number per group in the field deployment experiment. H= heat priming, K= chemical priming, M= mixed priming, C= control.


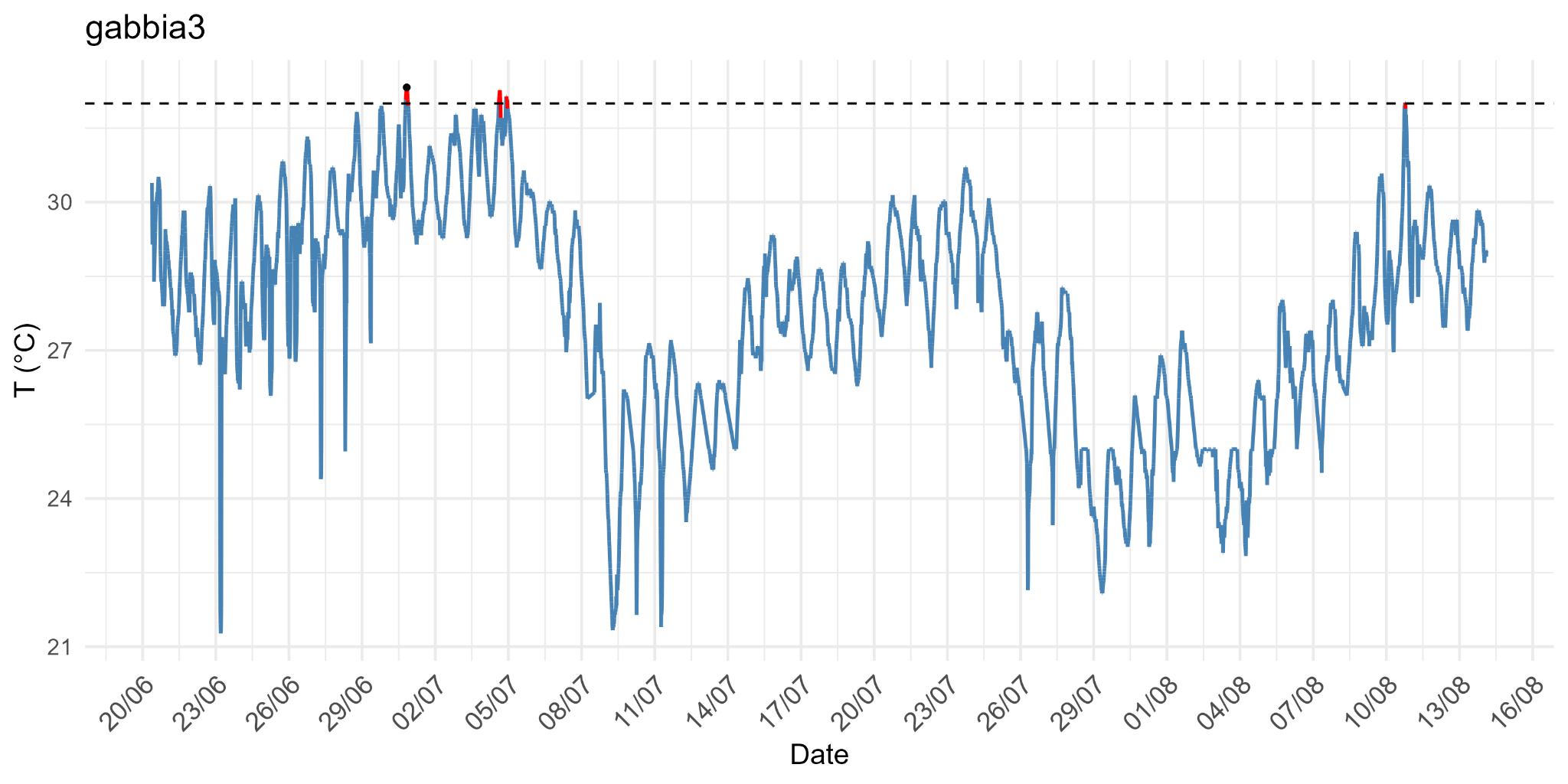


**Figure S2**: Temperatures registered at the deployment location during the field testing. The graph empathizes when the temperature exceeded 32°C (dashed line), corresponding in this case to two events persisting less than two days.


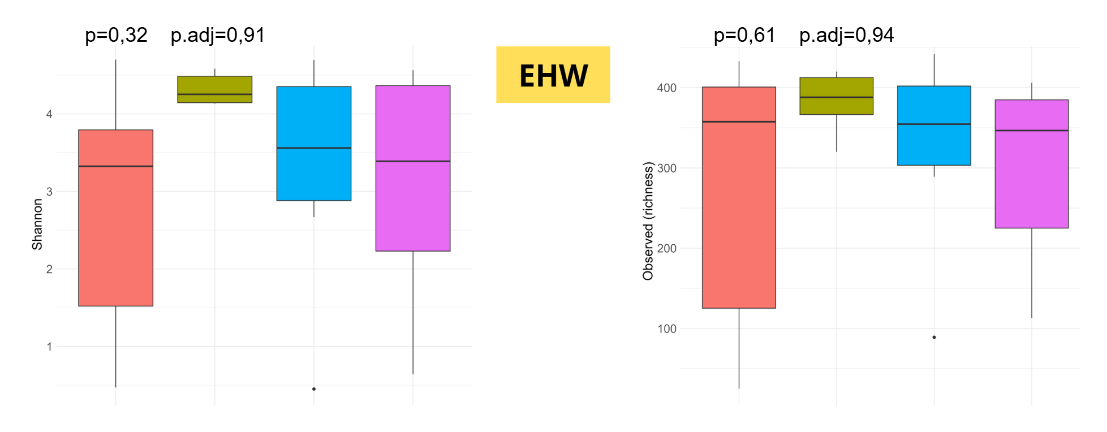

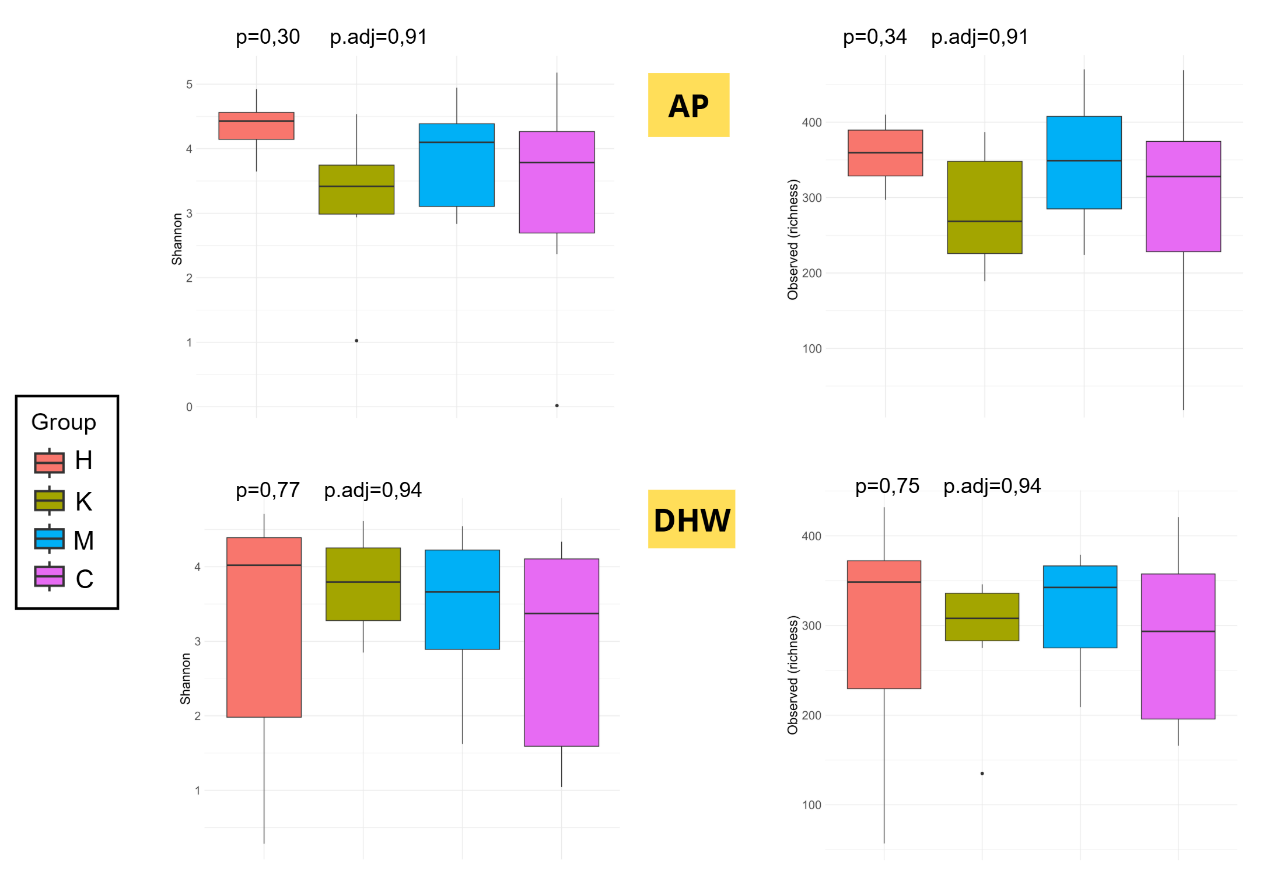


**Figure S3**: Boxplots of Obsterved Richness and Shannon indices for the digestive gland bacterial communities across experimental groups, at ASV level (H= heat priming, K= chemical priming, M= mixed priming, C= control) and phases (AP= after priming, DHW= during the heatwave, EHW= at the end of the heatwave). Global Kuskal-Wallis p-values and adjusted p-values are indicated.


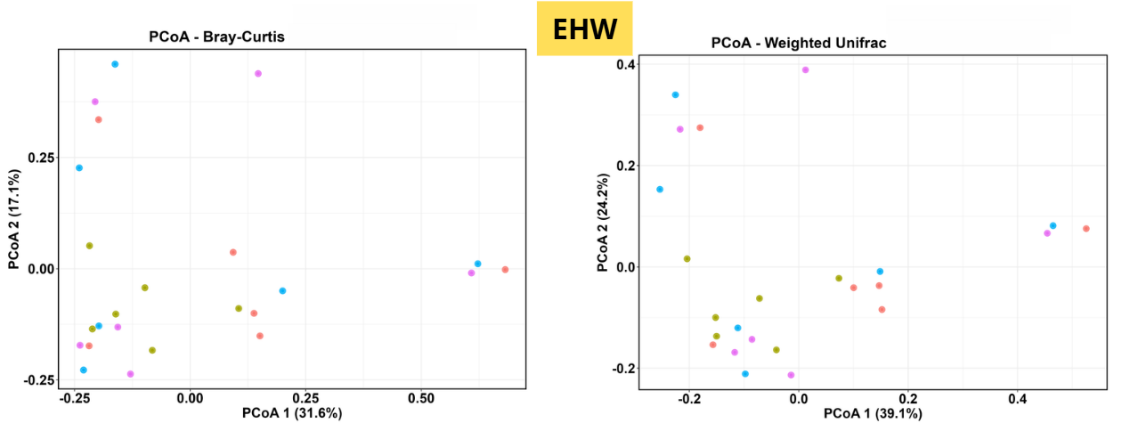

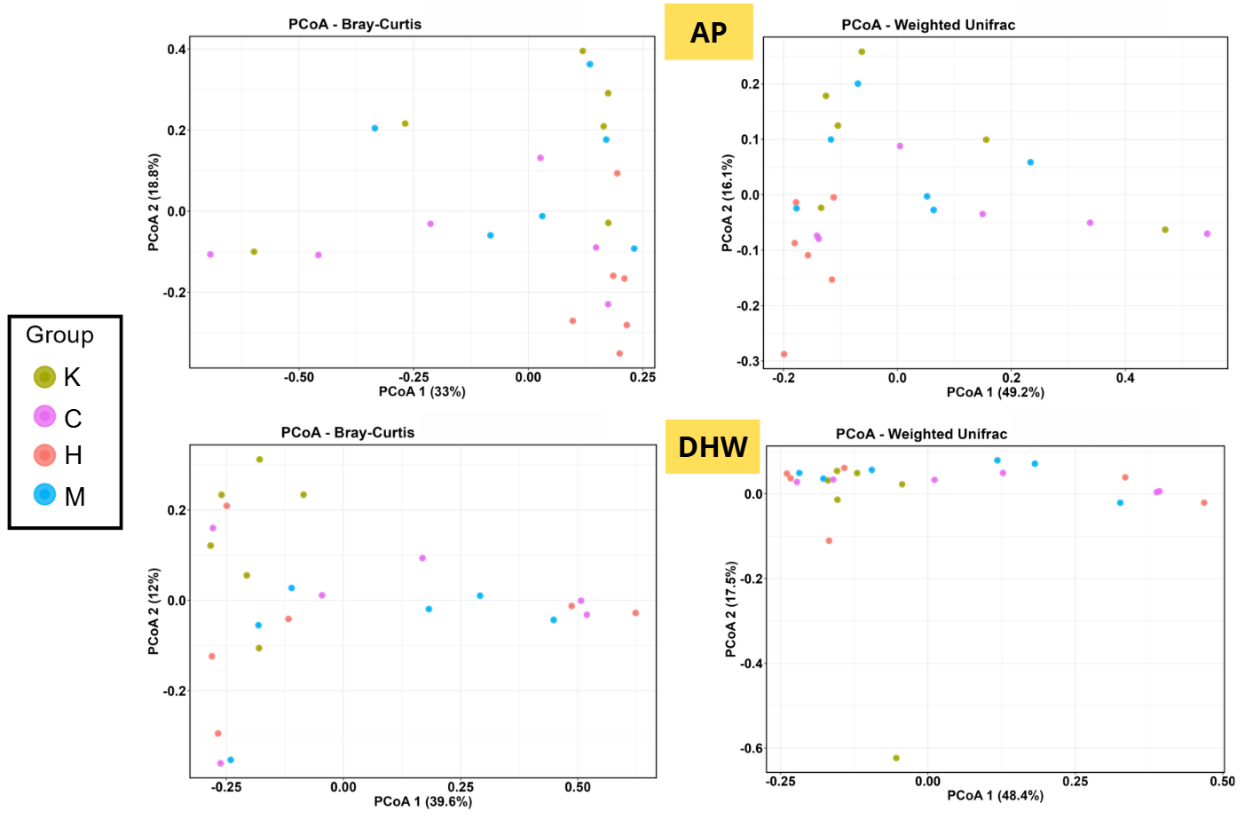


**Figure S4**: Principal Coordinates Analysis (PcoA) based on Bray-Curtis (left) and Unifrac (right) dissimilarity for the digestive gland bacterial communities across experimental groups, at ASV level (H= heat priming, K= chemical priming, M= mixed priming, C= control) and phases (AP= after priming, DHW= during the heatwave, EHW= at the end of the heatwave).


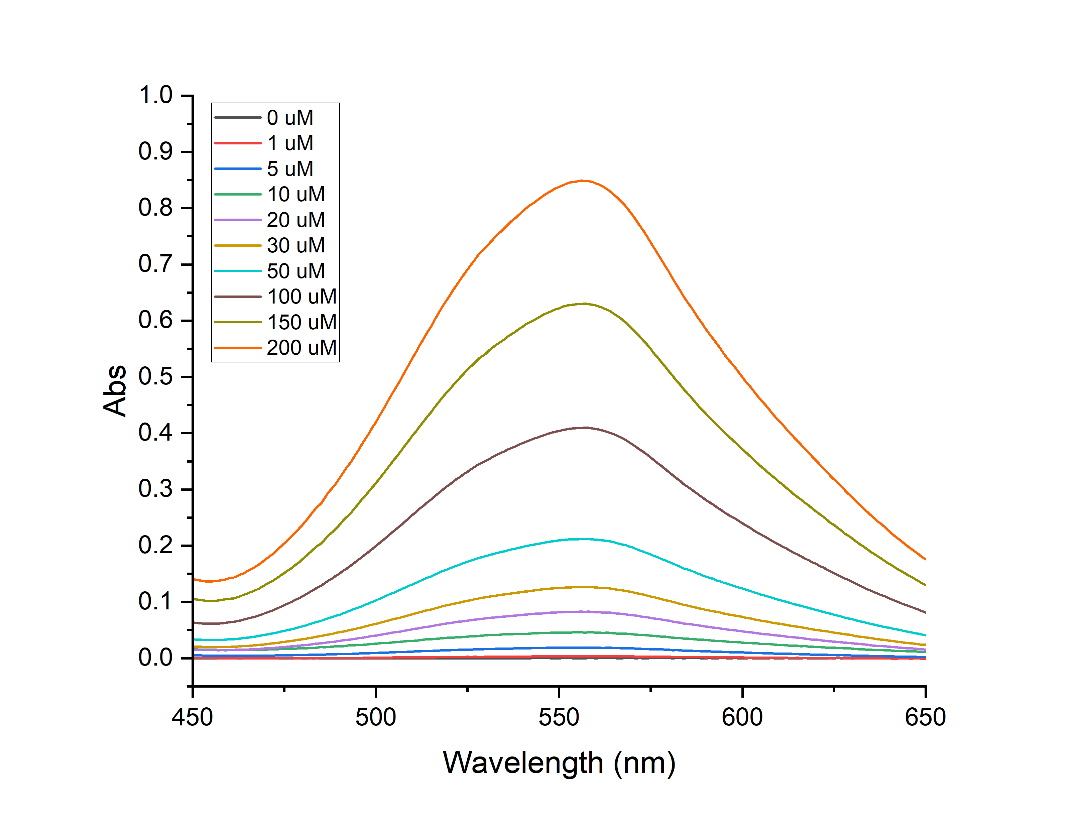


**Figure S5**: Absorbance spectra of the colored product obtained through the colorimetric assay described in the text, at different hydrogen peroxide concentrations. H2O2 standards ranged from 0 µM to 200 µM in marine water.


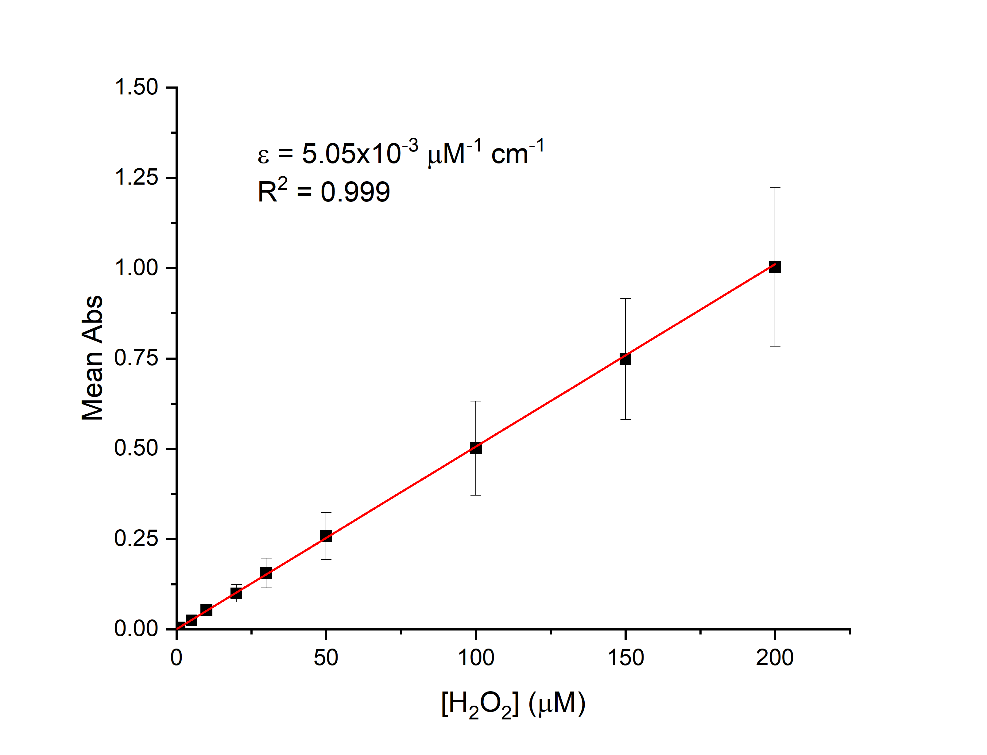


**Figure S6**: Calibration curve of H2O2 quantification through the spectrophotometric assay described in the text. Data resulted from means and standard deviations of three different replicates.


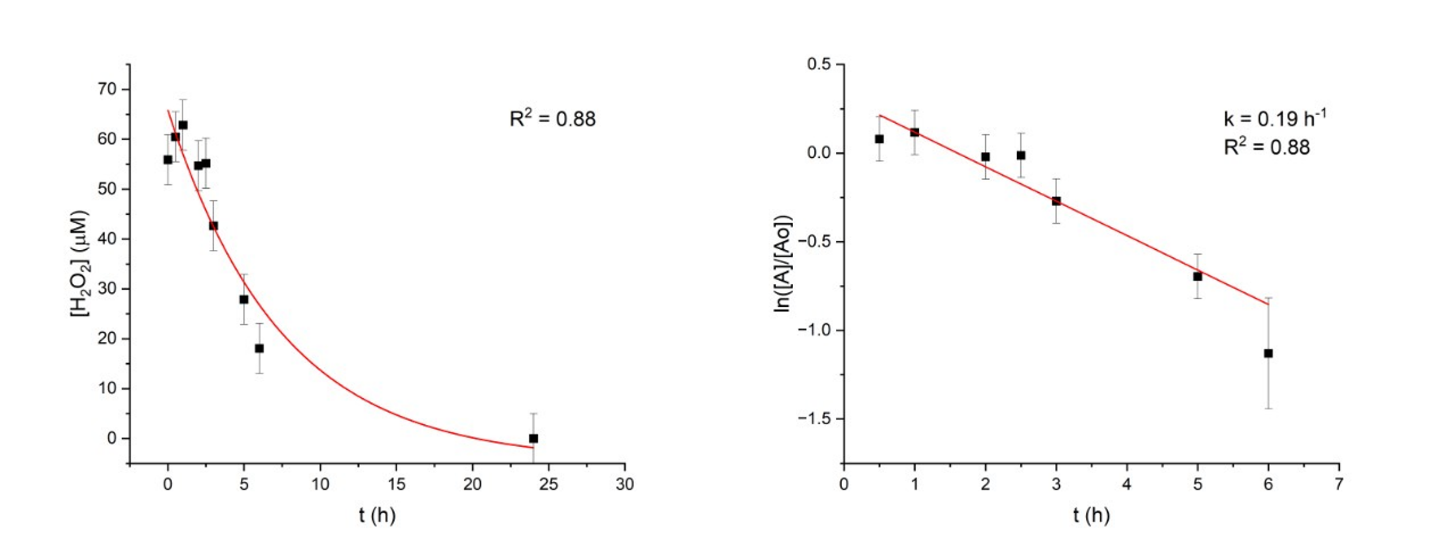


**Figure S7**: Left panel: Time-dependent evaluation of H2O2 decay in aquaria marine water, at room temperature. The quantification was made with the spectrophotometric assay described in the text. Right panel: linearized form of the H2O2 first order decay kinetics in marine water with clams, where [A] is the compound concentration at a defined time t, Ao is the initial compound concentration at time 0 min, and k is the rate constant. Data resulted from means and standard deviations of three different replicates.

**
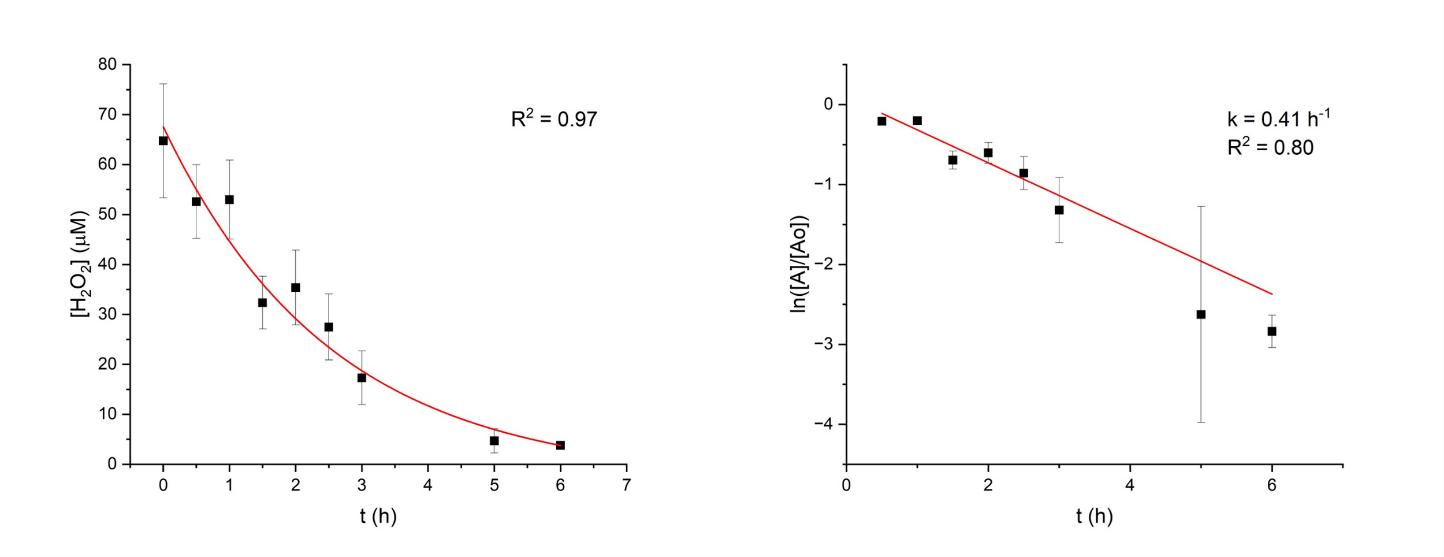
**

**Figure S8**: Left panel: Time-dependent evaluation of H2O2 concentration in marine water of aquaria hosting clams, at room temperature. The quantification was made with the spectrophotometric assay described in the text. Right panel: linearized form of the H2O2 first order decay kinetics in marine water with clams, where [A] is the compound concentration at a defined time t, Ao is the initial compound concentration at time 0 min, and k is the rate constant. Data resulted from means and standard deviations of three different replicates.


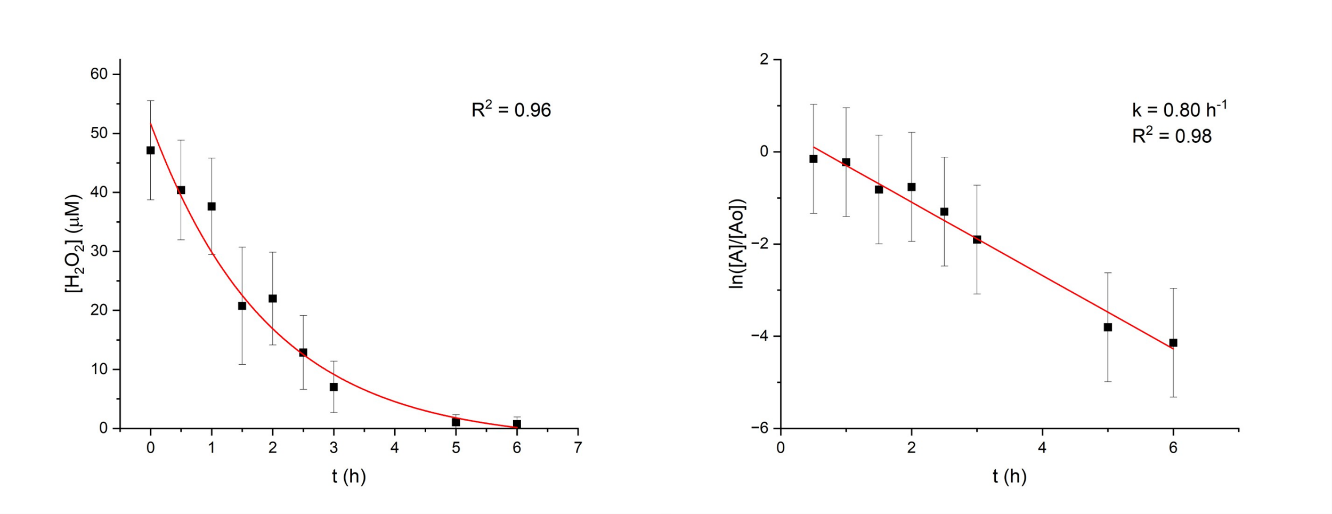


**Figure S9**: Left panel: Time-dependent evaluation of H2O2 concentration in marine water of aquaria hosting clams, at 30°C temperature. The quantification was made with the spectrophotometric assay described in the text. Right panel: linearized form of the H2O2 first order decay kinetics in marine water with clams, where [A] is the compound concentration at a defined time t, Ao is the initial compound concentration at time 0 min, and k is the rate constant. Data resulted from means and standard deviations of three different replicates.


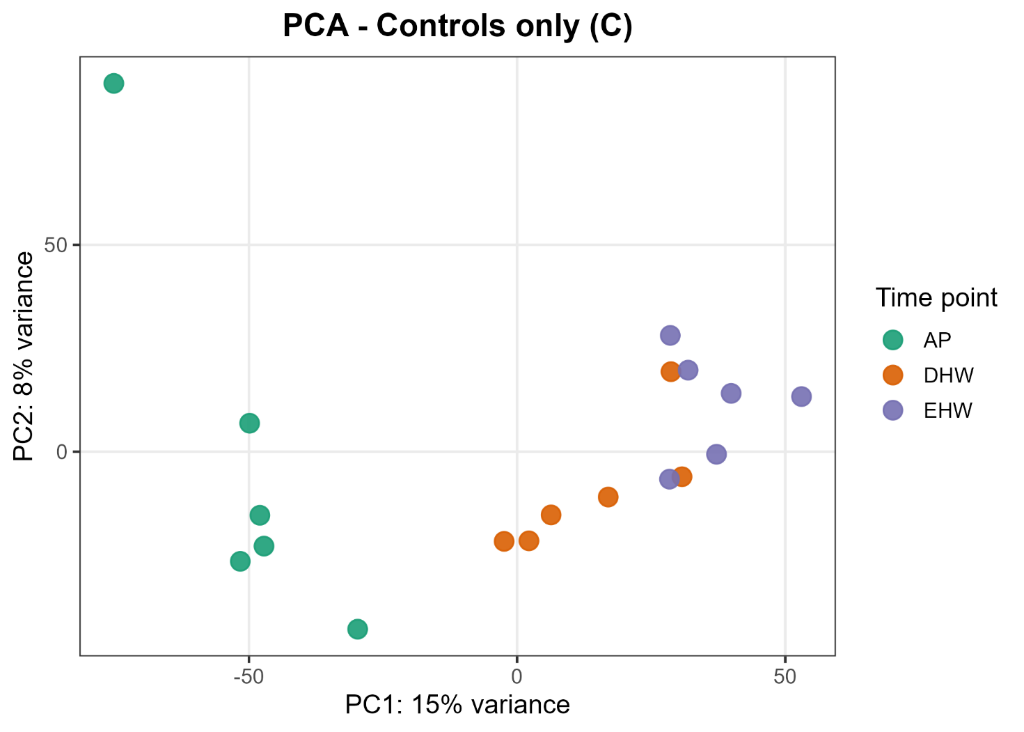


**Figure S10:** Principal component analysis (PCA) of gene expression profiles in control (non-primed) clams across experimental time points. Samples are colored by time point: after priming (AP), during heatwave exposure (DHW), and at the end of the heatwave (EHW).

REFERENCES

(1) Di Tommaso, P.; Chatzou, M.; Floden, E. W.; Barja, P. P.; Palumbo, E.; Notredame, C. Nextflow Enables Reproducible Computational Workflows. *Nat. Biotechnol.* **2017**, *35* (4), 316–319. https://doi.org/10.1038/nbt.3820.

(2) Risso, D.; Ngai, J.; Speed, T. P.; Dudoit, S. Normalization of RNA-Seq Data Using Factor Analysis of Control Genes or Samples. *Nat. Biotechnol.* **2014**, *32* (9), 896–902. https://doi.org/10.1038/nbt.2931.

(3) Wu, T.; Hu, E.; Xu, S.; Chen, M.; Guo, P.; Dai, Z.; Feng, T.; Zhou, L.; Tang, W.; Zhan, L.; Fu, X.; Liu, S.; Bo, X.; Yu, G. clusterProfiler 4.0: A Universal Enrichment Tool for Interpreting Omics Data. *The Innovation* **2021**, *2* (3), 100141. https://doi.org/10.1016/j.xinn.2021.100141.

(4) Yu, G.; Wang, L.-G.; Yan, G.-R.; He, Q.-Y. DOSE: An R/Bioconductor Package for Disease Ontology Semantic and Enrichment Analysis. *Bioinformatics* **2015**, *31* (4), 608–609. https://doi.org/10.1093/bioinformatics/btu684.

(5) McMurdie, P. J.; Holmes, S. Phyloseq: An R Package for Reproducible Interactive Analysis and Graphics of Microbiome Census Data. *PLoS ONE* **2013**, *8* (4), e61217. https://doi.org/10.1371/journal.pone.0061217.

(6) Dixon, P. VEGAN, a Package of R Functions for Community Ecology. *J. Veg. Sci.* **2003**, *14* (6), 927–930. https://doi.org/10.1111/j.1654-1103.2003.tb02228.x.

(7) Love, M. I.; Huber, W.; Anders, S. Moderated Estimation of Fold Change and Dispersion for RNA-Seq Data with DESeq2. *Genome Biol.* **2014**, *15* (12), 550. https://doi.org/10.1186/s13059-014-0550-8.

(8) Stevanato, R.; Mondovi’, B.; Sabatini, S.; Rigo, A. Spectrophotometric Assay for Total Polyamines by Immobilized Amine Oxidases. *Anal. Chim. Acta* **1990**, *237*, 391–397. https://doi.org/10.1016/S0003-2670(00)83942-2.
